# Soybean RNA interference lines silenced for eIF4E show broad potyvirus resistance

**DOI:** 10.1111/mpp.12897

**Published:** 2019-12-20

**Authors:** Le Gao, Jinyan Luo, Xueni Ding, Tao Wang, Ting Hu, Puwen Song, Rui Zhai, Hongyun Zhang, Kai Zhang, Kai Li, Haijian Zhi

**Affiliations:** ^1^ National Center for Soybean Improvement Nanjing Agricultural University Nanjing China; ^2^ College of Agronomy and Biotechnology China Agricultural University Beijing China; ^3^ Institute of Cereal and Oil Crops Handan Academy of Agricultural Sciences Handan China

**Keywords:** *Agrobacterium*‐mediated transformation, broad‐spectrum resistance, eIF4E, *Potyvirus*, RNA interference, soybean, *Soybean mosaic virus*

## Abstract

*Soybean mosaic virus* (SMV), a potyvirus, is the most prevalent and destructive viral pathogen in soybean‐planting regions of China. Moreover, other potyviruses, including bean common mosaic virus (BCMV) and watermelon mosaic virus (WMV), also threaten soybean farming. The eukaryotic translation initiation factor 4E (eIF4E) plays a critical role in controlling resistance/susceptibility to potyviruses in plants. In the present study, much higher SMV‐induced *eIF4E1* expression levels were detected in a susceptible soybean cultivar when compared with a resistant cultivar, suggesting the involvement of *eIF4E1* in the response to SMV by the susceptible cultivar. Yeast two‐hybrid and bimolecular fluorescence complementation assays showed that soybean eIF4E1 interacted with SMV VPg in the nucleus and with SMV NIa‐Pro/NIb in the cytoplasm, revealing the involvement of VPg, NIa‐Pro, and NIb in SMV infection and multiplication. Furthermore, transgenic soybeans silenced for eIF4E were produced using an RNA interference approach. Through monitoring for viral symptoms and viral titers, robust and broad‐spectrum resistance was confirmed against five SMV strains (SC3/7/15/18 and SMV‐R), BCMV, and WMV in the transgenic plants. Our findings represent fresh insights for investigating the mechanism underlying eIF4E‐mediated resistance in soybean and also suggest an effective alternative for breeding soybean with broad‐spectrum viral resistance.

## Graphical abstract

Our findings confirm that soybean eIF4E is the susceptibility factor for soybean mosaic virus, and broad‐spectrum potyvirus resistance is developed in transgenic soybean via RNA interference approach.

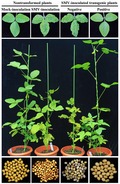

## 1 INTRODUCTION

Soybean (*Glycine max*), one of the most important crops worldwide, is indispensable to the human diet owing to its high content of high‐quality vegetable oil and protein (Gao *et al*., 2015a). However, plant pathogens are a major constraint to agricultural production (Dodds, 2010), and soybean growth is often impeded by a multitude of pathogens, including oomycetes, nematodes, fungi, bacteria, and viruses, which are responsible for significant economic losses annually (Liu *et al*., 2016; Whitham *et al*., 2016). Among these, *soybean mosaic virus* (SMV) is the most widespread and devastating viral pathogen in soybean‐growing areas, resulting in serious yield reductions and seed quality deterioration (Hill and Whitham, 2014; Hajimorad *et al*., 2018). Yield losses are usually reported to be approximately 8–35% (Hill and Whitham, 2014); however, losses of more than 50% and even total crop failure have been documented during severe outbreaks (Liao *et al*., 2002). SMV originates from SMV‐infected seeds and is nonpersistently transmitted by more than 30 different migratory aphid species, within and among soybean fields (Steinlage *et al*., 2002). Symptoms induced by SMV infection include mosaic patterns, chlorosis, rugosity, curling, and necrosis of soybean leaves, subsequently leading to plant dwarfing and seed discoloration (seed coat mottling), which significantly reduces the commercial value of soybean seeds (Kim *et al*., 2016; Zhang *et al*., 2011). The tremendous damage suffered from SMV necessitates the introduction of viral resistance in soybean crops for improving soybean production and productivity in China (Gao *et al*., 2015b, 2018).

*Soybean mosaic virus* is a member of the largest and most successful genus of plant pathogenic viruses, *Potyvirus*, within the family *Potyviridae* (Adams *et al*., 2005; Luan *et al*., 2016). Similar to other potyviruses, the genome of SMV is a monopartite, single‐stranded, positive‐sense RNA molecule of approximately 10 kb, harboring a viral genome‐linked protein (VPg) covalently attached to the 5′ terminus and a poly(A) tail at the 3′ end (Gagarinova *et al*., 2008; Hajimorad *et al*., 2018). The viral genome contains two open reading frames (ORF) encoding 11 mature multifunctional proteins, namely protein 1 (P1), helper component‐proteinase (HC‐Pro), protein 3 (P3), pretty interesting *Potyviridae* ORF (P3N‐PIPO), six kilodalton 1 (6K1), cylindrical inclusion protein (CI), six kilodalton 2 (6K2), VPg, nuclear inclusion a‐proteinase (NIa‐Pro), nuclear inclusion b (NIb), and coat protein (CP) (Chung *et al*., 2008; Gagarinova *et al*., 2008). Furthermore, based on their differential responses and pathogenicity to soybean plants, numerous SMV isolates have been grouped into seven strains (G1–G7) in the United States (Cho and Goodman, 1979) and into 22 strains (SC1–SC22) in China (Li *et al*., 2010). Additionally, a novel recombinant SMV strain (SMV‐R), which likely originated from an interspecific recombination event between SMV and bean common mosaic virus (BCMV) or a BCMV‐like virus, has been identified in China (Yang *et al*., 2011, 2014).

The use of naturally occurring host resistance is the most economical, effective, and eco‐friendly approach for protecting against plant pathogens and preventing crop yield losses in agricultural practices (Kang *et al*., 2005; Maule *et al*., 2007). Resistance genes can be categorized as dominant or recessive, based on their inheritance; interestingly, dominant resistance genes predominantly confer resistance against bacteria and fungi, while recessive resistance appears to be more frequently found for viruses than for other plant pathogens (Diaz‐Pendon *et al*., 2004; Truniger and Aranda, 2009; Wang and Krishnaswamy, 2012; Chandrasekaran *et al*., 2016). More specifically, genes conferring recessive resistance against potyviruses are much more frequent than those against other viruses, and potyviral resistance is often not restricted to a single potyvirus (Provvidenti and Hampton, 1992; Ruffel *et al*., 2002).

Host factors are essential in the viral infection cycle and therefore recessive resistance against viruses can be induced if one or more host factors are absent or mutated via a mechanism known as resistance by loss of susceptibility (Charron *et al*., 2008; Bastet *et al*., 2017). Natural recessive resistance genes involved in the plant–virus pathosystem have been successfully exploited in diverse crop species, including pepper (*Capsicum annuum*), lettuce (*Lactuca sativa*), pea (*Pisum sativum*), common bean (*Phaseolus vulgaris*), barley (*Hordeum vulgare*), tomato (*Solanum lycopersicum*), melon (*Cucumis melo*), Chinese cabbage (*Brassica rapa*), and rice (*Oryza sativa*) (Ruffel *et al*., 2002; Nicaise *et al*., 2003; Gao *et al*., 2004; Kanyuka *et al*., 2005; Albar *et al*., 2006; Nieto *et al*., 2006; Naderpour *et al*., 2010; Kim *et al*., 2013b; Gauffier *et al*., 2016), and the majority of these genes are associated with the eukaryotic translation initiation factor 4E (eIF4E) or its isoform, eIF(iso)4E.

eIF4E is a cap‐binding protein that specifically interacts with the 5′‐terminal cap structure of mRNA (m^7^GpppN) and plays a critical role in initiating mRNA translation and regulating protein synthesis (Wang and Krishnaswamy, 2012; Sanfaçon, 2015). As potyviral VPg substitutes for functions of the mRNA cap structure in initiating viral translation (Moury *et al*., 2014), eIF4E has been identified as the major susceptibility factor for potyviruses (Robaglia and Caranta, 2006; Bastet *et al*., 2017). eIF4E‐mediated resistance has been developed as a novel strategy for rendering hosts nonpermissive to viral infection, and it has been successfully shown in tomato (Mazier *et al*., 2011), melon (Rodríguez‐Hernández *et al*., 2012), plum (*Prunus domestica*) (Wang *et al*., 2013), and peanut (*Arachis hypogaea*) (Xu *et al*., 2017) using RNA interference (RNAi). However, eIF4E‐mediated viral resistance has not yet been employed in genetically engineered soybean.

Previous studies have shown that eIF4E and its isoform eIF(iso)4E can be selectively recruited in various plant–potyvirus pairs (Duprat *et al*., 2002; Lellis *et al*., 2002; Sato *et al*., 2005; Estevan *et al*., 2014). eIF4E belongs to a multigene family, of which four genes, that is, *eIF4E1* (accession no. EU912426), *eIF4E2* (accession no. XM_003546012), *eIF(iso)4E1* (accession no. XM_003535948), and *eIF(iso)4E2* (accession no. BT098172), have been reported in soybean (Wang *et al*., 2013; Xu *et al*., 2017). Our previous research (Zhang, 2012) focused on *eIF4E1* and *eIF(iso)4E1*, with a total of 208 soybean cultivars being used for SMV resistance assessment and 17 cultivars being identified as SMV resistant (Table S7). Further analyses on these 17 resistant cultivars proved that, compared with the soybean cultivar Nannong 1138–2 (highly susceptible host), five resistant cultivars harbored mutated eIF4E1s (Table S8 and Text S1), of which four were unable to interact with SMV VPg in the yeast two‐hybrid (Y2H) screen system (Table S8). Furthermore, all eIF(iso)4E1s from the 17 resistant cultivars were the same and identical to that of Nannong 1138–2 (Text S1). Consequently, we speculated that eIF4E, rather than eIF(iso)4E, might play the leading role in the soybean–SMV pathosystem. Thus, in the present study, we focused on *eIF4E1*.

Considering the unique status of eIF4E, both as a crucial regulator of cellular metabolism and a controller of resistance/susceptibility to potyviruses, we conducted experiments to identify spatiotemporal expression patterns of *eIF4E1* in soybean, to analyse subcellular localization in *Nicotiana benthamiana*, and to determine its protein–protein interactions with SMV. Furthermore, using RNAi via a cotyledonary node–*Agrobacterium*‐mediated transformation system, transgenic soybean plants expressing the transgene construct of inverted repeat‐*eIF4E1i* fragments, which were able to form the RNA hairpin structure inducing specific post‐transcriptional gene silencing of *eIF4E1*, were developed. Robust and broad‐spectrum resistance against multiple SMV strains and two additional potyviruses, namely BCMV and watermelon mosaic virus (WMV), was observed in transgenic soybeans and was confirmed by monitoring for viral symptoms and viral titers. Results from this study provide fresh insights for investigating the molecular basis of eIF4E‐mediated resistance in soybean, and may indicate an alternative strategy for breeding soybean resistant to SMV and other potyviruses.

## 2 RESULTS

### 2.1 Spatiotemporal expression analysis of soybean *eIF4E1*

**Figure 1:**
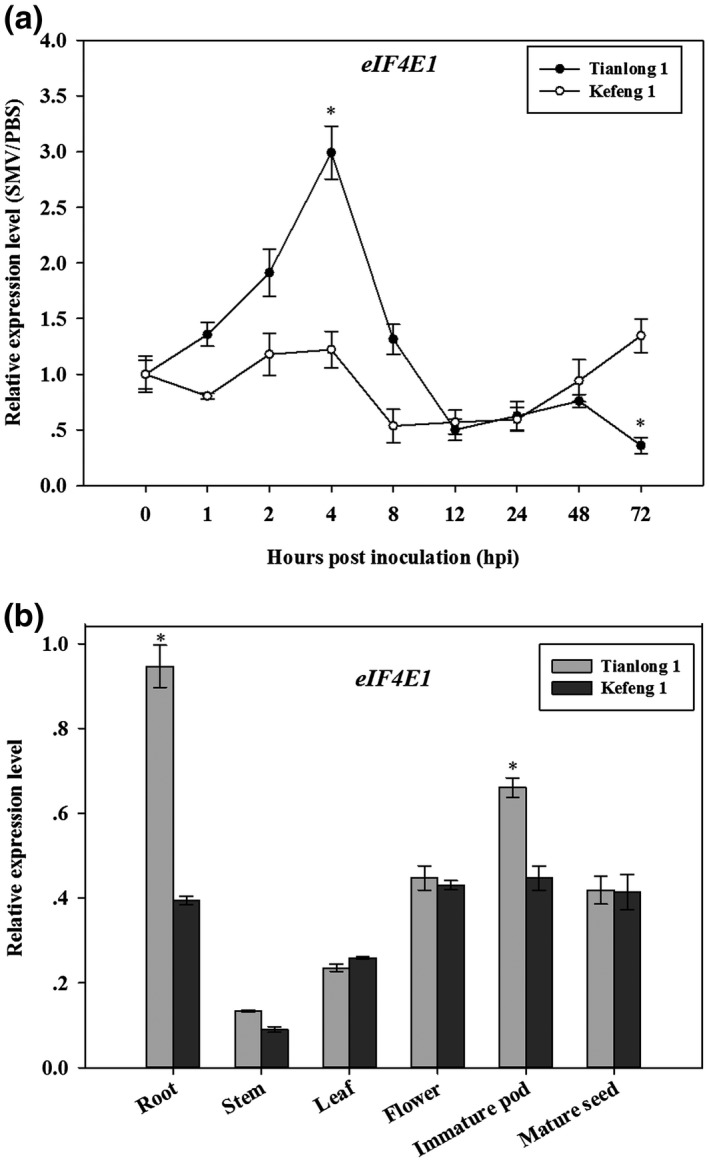
Spatiotemporal expression analysis of *eIF4E1* in soybean cultivars Tianlong 1 and Kefeng 1 using RT‐qPCR. (a) Temporal expression profiles of *eIF4E1* in the inoculated leaves after challenge with *soybean mosaic virus* (SMV) strain SC3 at different time points. Data were calibrated using phosphate‐buffered saline (PBS)‐inoculated controls. (b) Spatial expression profiles of *eIF4E1* in various healthy tissues. Error bars indicate *SD* (*n* = 3). Asterisks indicate significant difference between susceptible and resistant plants at the corresponding time points and tissues, *t* test, *p* < .001. Results are representative of three independent experiments

In case of temporal responses of *eIF4E1* to SMV infection, the relative expression levels in Tianlong 1 (SMV susceptible) showed obvious up‐ and down‐regulation patterns before and after 4 hr post‐inoculation (hpi), respectively, exhibiting maximum expression by approximately 3.0‐fold at 4 hpi (Figure 1a). In Kefeng 1 (SMV resistant), *eIF4E1* expression levels remained relatively stable and were evidently lower than those of Tianlong 1 at the overall level (Figure 1a). Compared with Kefeng 1, the substantially higher *eIF4E1* expression levels induced by SMV in Tianlong 1 indicated the involvement of *eIF4E1* in SMV responses of the susceptible cultivar.

Regarding the spatial expression patterns of *eIF4E1*, we found that transcript levels in Tianlong 1 varied in different healthy tissues, and the highest and lowest values were observed in the root and stem, respectively (Figure 1b). However, *eIF4E1* transcript levels in Kefeng 1 were similar in the root, flowers, immature pods, and mature seeds, and the lowest value was recorded in the stem (Figure 1b). High *eIF4E1* transcript levels observed in the root and immature pods of Tianlong 1 (Figure 1b) demonstrated the up‐regulated expression pattern of *eIF4E1* in young tissues, which was consistent with the results of previous studies in plum and peanut (Wang *et al*., 2013; Xu *et al*., 2017).

### 2.2 Subcellular localization of soybean eIF4E1 and analysis of protein–protein interaction with SMV

**Figure 2:**
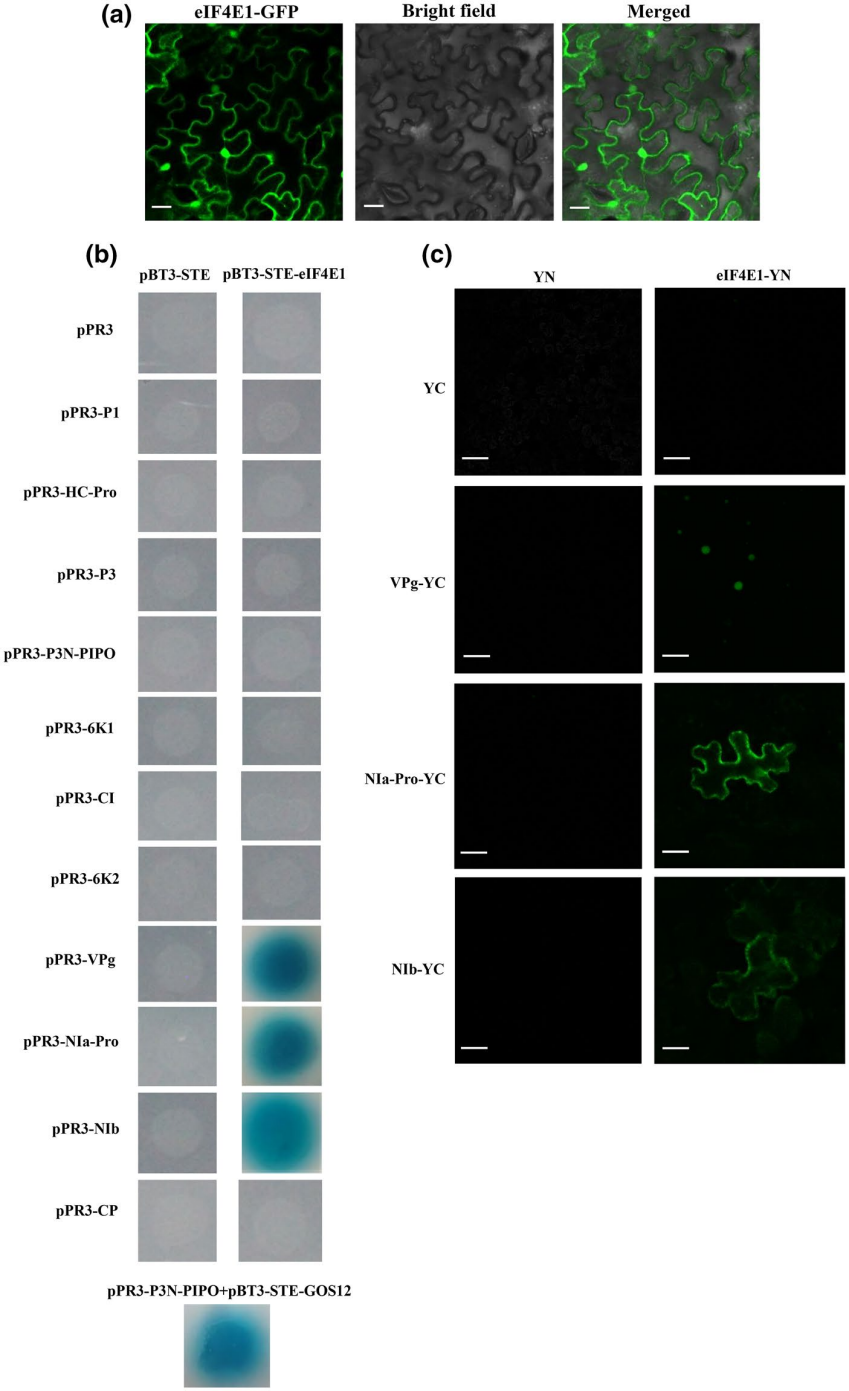
Subcellular localization of soybean eIF4E1 and analysis of protein–protein interaction with *soybean mosaic virus* (SMV). (a) Subcellular localization in *Nicotiana benthamiana* leaf cells. Soybean eIF4E1 fused with green fluorescent protein (GFP) was agroinfiltrated into leaves of 4‐week‐old *N. benthamiana*. Scale bars = 20 μm. (b) Yeast two‐hybrid screen system. Yeast co‐transformants were identified on selective quadruple dropout medium *SD*/−Leu/−Trp/−Ade/−His/+X‐α‐Gal with blue color staining. Yeast containing pBT3‐STE + pPR3, pBT3‐STE‐eIF4E1 + pPR3, or pBT3‐STE + pPR3‐SMV served as negative controls. Yeast cells co‐transformed with pPR3‐P3N‐PIPO + pBT3‐STE‐GOS12 were used as positive control. (c) Bimolecular fluorescence complementation assay. eIF4E1‐YN and SMV‐YC were co‐agroinfiltrated into leaves of 4‐week‐old *N. benthamiana*. Interactions between YN and YC, YN and SMV‐YC, and eIF4E1‐YN and YC were used as negative controls. Scale bars = 20 μm

To examine the intracellular distribution of soybean eIF4E1 in planta, eIF4E1 was fused with green fluorescent protein (GFP) and transiently expressed in *N. benthamiana*. The results suggested that eIF4E1 was present in both the nucleus and cytoplasm (Figure 2a). As shown by Y2H analysis (Figure 2b), eIF4E1 may interact with three SMV proteins, including VPg, NIa‐Pro, and NIb, while no interactions were detected between eIF4E1 and the other eight SMV proteins (Figure 2b). The results of Y2H analysis were further confirmed by the bimolecular fluorescence complementation (BiFC) assay. A nucleus signal was observed with eIF4E1–YN and VPg–YC combinations, and cytoplasm signals were recorded with eIF4E1–YN and NIa‐Pro/NIb–YC combinations (Figure 2c). As expected, no fluorescence signals were detected in the negative controls (Figure 2c). In combination, these results indicated that eIF4E1 interacted with VPg in the nucleus, and with NIa‐Pro/NIb in the cytoplasm, revealing the involvement of VPg, NIa‐Pro, and NIb in SMV infection and multiplication.

### 2.3 Generation of transgenic soybean plants silenced for eIF4E1

**Figure 3:**
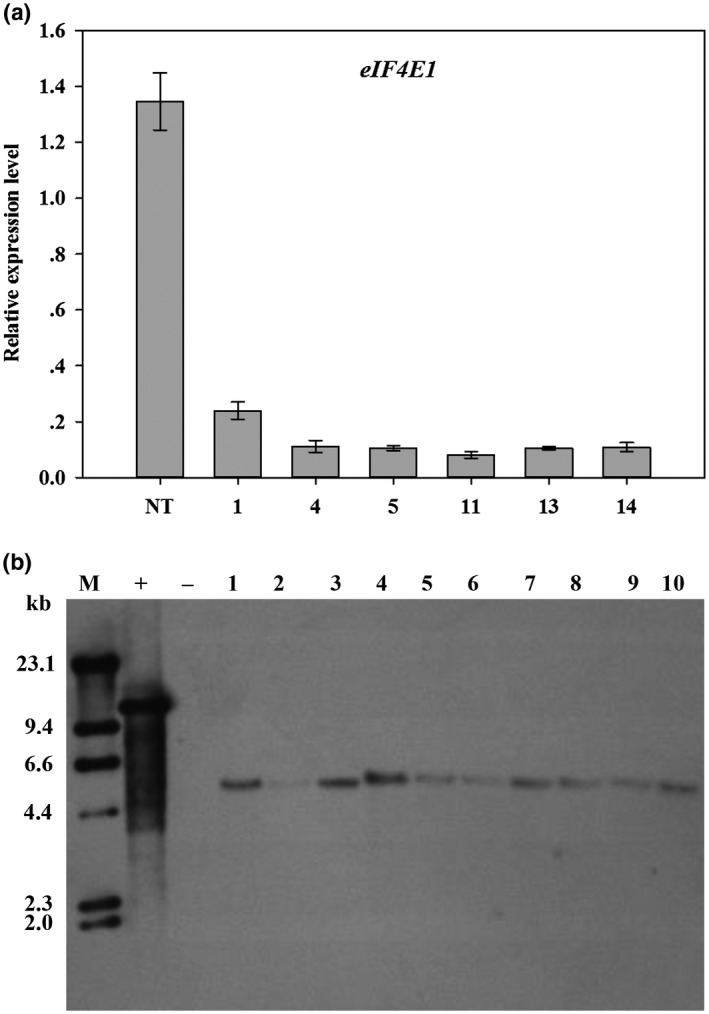
RT‐qPCR and Southern blot analyses of transgenic soybean plants. (a) RT‐qPCR detection of relative expression levels of *eIF4E1* in positive T_0_ plants. The *y* axis indicates *eIF4E1* transcript levels. The *x* axis indicates T_0_ and nontransformed (NT) plants. Results are representative of three independent experiments with error bars indicating *SD* (*n* = 3). (b) Southern blot hybridization analysis in T_1_ generation derived from T_0_ line 1. Total genomic DNA (c.30 μg) was digested with *Hin*dIII and hybridized with a *bar* probe (Figure S1) labelled with DIG. M, DNA molecular size; +, pB7GWIWG2(II)‐*eIF4E1i* vector used as positive control; −, genomic DNA of nontransformed soybean plants used as negative control; 1–10 represent transgene‐positive T_1_ plants

An RNAi strategy was employed to determine the role of soybean eIF4E1 in SMV infection, and 31 positive T_0_ plants were developed (Table S2). The silencing effect was assessed by quantitative real‐time reverse transcription polymerase chain reaction (RT‐qPCR) analysis of *eIF4E1* (primer 4 in Table S1) transcript levels in T_0_ plants. Significant reductions (approximately 80–90%) in *eIF4E1* transcript accumulation were detected in six randomly selected T_0_ plants when compared with that in nontransformed plants (Figure 3a), indicating that the silencing strategy was efficient.

Southern blot analysis was performed, and 10 T_1_ plants derived from T_0_ line 1 (Table 1) exhibited the same integration pattern (single copy of T‐DNA) in the soybean genome. As expected, all bands were greater than 3.66 kb in size (Figure 3b), which was greater than the fragment between the left border and the unique *Hin*dIII site (Figure S1), and the hybridization signal was not detected in nontransformed plants. The single T‐DNA insertion strongly suggested stable heritability, and two of these 10 T_1_ plants (Table 1) were selected for propagating homozygous progenies for further analyses.

**Table 1:** Classification of responses of T_1_ and T_2_ soybean plants to SMV strain SC3

T_1_ generation^a^					T_2_ generation^a^				
T_0_ line no.	No. of T_1_ plants evaluated	Highly resistant^b^	Mildly resistant^c^	Susceptible^d^	T_1_ line no.	No. of T_2_ plants evaluated	Highly resistant^b^	Mildly resistant^c^	Susceptible^d^
1	25	25	0	0	1–1	26	20	6	0
3	6	4	2	0	1–16	16	13	3	0
4	8	2	5	1	Total	42	33 (78.6%)	9 (21.4%)	0 (0)
5	27	4	6	17					
6	10	6	1	3					
7	3	0	2	1					
11	10	0	4	6					
12	2	2	0	0					
13	12	2	6	4					
14	9	2	3	4					
16	1	0	1	0					
18	2	0	2	0					
19	8	0	5	3					
20	4	1	2	1					
21	16	1	14	1					
22	1	0	1	0					
31	3	1	2	0					
40	1	0	1	0					
Total	148	50 (33.8%)	57 (38.5%)	41 (27.7%)					

Note

SMV, *soybean mosaic virus*.

a Twenty nontransformed plants were evaluated and they were all susceptible.

b Highly resistant plants with no visible viral symptoms.

c Mildly resistant plants with delayed appearance of viral symptoms or symptoms lighter than those of nontransformed controls.

d Susceptible plants with viral symptoms identical to those of nontransformed controls.

### 2.4 Robust SMV resistance in T_1_ and T_2_ generations

**Figure 4:**
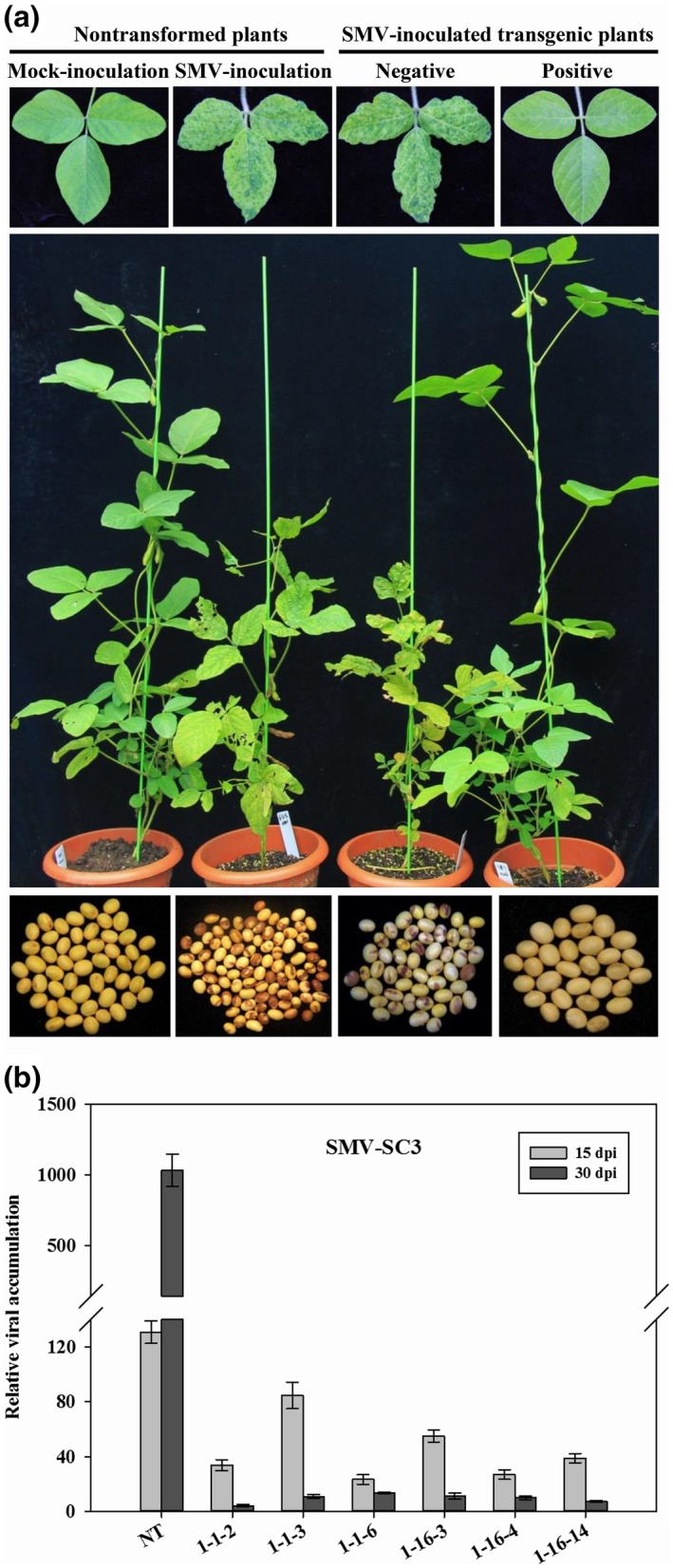
*Soybean mosaic virus* (SMV) resistance assessments in T_1_/T_2_ generations. (a) Appearance of symptoms in T_1_ soybean plants after challenge with SMV strain SC3. Mock‐inoculated and SMV‐inoculated nontransformed plants were used as controls. (b) RT‐qPCR detection of systemic virus accumulation in leaves of T_2_ plants derived from T_0_ line 1 after challenge with SMV strain SC3. The *y* axis indicates SMV transcript levels at 15 and 30 days post‐inoculation (dpi). The *x* axis indicates T_2_ and nontransformed (NT) plants. Results are representative of three independent experiments with error bars indicating *SD* (*n* = 3)

One hundred and forty‐eight T_1_ soybean plants from 18 independent T_0_ lines and 42 T_2_ plants from T_0_ line 1 were inoculated with SMV strain SC3 for resistance evaluation, and the various responses are outlined in Table 1. In general, SMV resistance was improved in T_1_ generation, in which 50 (33.8%) highly resistant, 57 (38.5%) mildly resistant, and 41 (27.7%) susceptible plants were identified (Table 1). Of all the T_0_ lines, T_0_ line 1 presented the best SMV resistance, with all T_1_ progenies being highly resistant (Table 1). Hence, two T_1_ plants (nos. 1–1 and 1–16, Table 1) derived from T_0_ line 1 were selected for generating T_2_–T_4_ progenies for further analyses. In the T_2_ generation, 33 highly resistant plants were confirmed, with a percentage of up to 78.6%, and no susceptible plants were found (Table 1). Following the SMV challenge, nontransformed and negative T_1_ plants exhibited typical mosaic leaves, remarkably dwarf plant phenotypes, and severe seed discoloration (Figure 4a). However, resistant T_1_ plants were symptomless, exhibited healthy growth, and produced clean seeds, similar to those of the mock control (Figure 4a). Moreover, unlike nontransformed plants, which produced 84.65% mottled seeds, only 30.89% of the seeds harvested from T_1_ lines were mottled, and seed coat mottling in T_2_–T_4_ lines was almost completely eliminated (Table S3).

Furthermore, six highly resistant T_2_ plants were randomly selected for RT‐qPCR detection, and all 42 T_2_ plants were used for double antibody sandwich enzyme‐linked immunosorbent assay (DAS‐ELISA) testing. Contrary to the nontransformed plants, in which virus accumulation increased considerably from 15 to 30 days post‐inoculation (dpi) (Figure 4b), the SMV content in T_2_ plants was markedly reduced and was evidently lower than that of nontransformed plants at both time points (Figure 4b). In addition, particularly at 30 dpi, T_2_ plants exhibited negligible viral content (Figure 4b). In the DAS‐ELISA analysis, only three T_2_ plants were identified as SMV susceptible, and viral titers of the other T_2_ plants were below the detection limits (Table S4).

These results proved that robust SMV resistance can be achieved by silencing soybean *eIF4E1* using RNAi, implying that soybean *eIF4E1* acted as a susceptibility factor for SMV infection.

### 2.5 Broad‐spectrum resistance against multiple potyviruses in T_3_ and T_4_ generations

**Figure 5:**
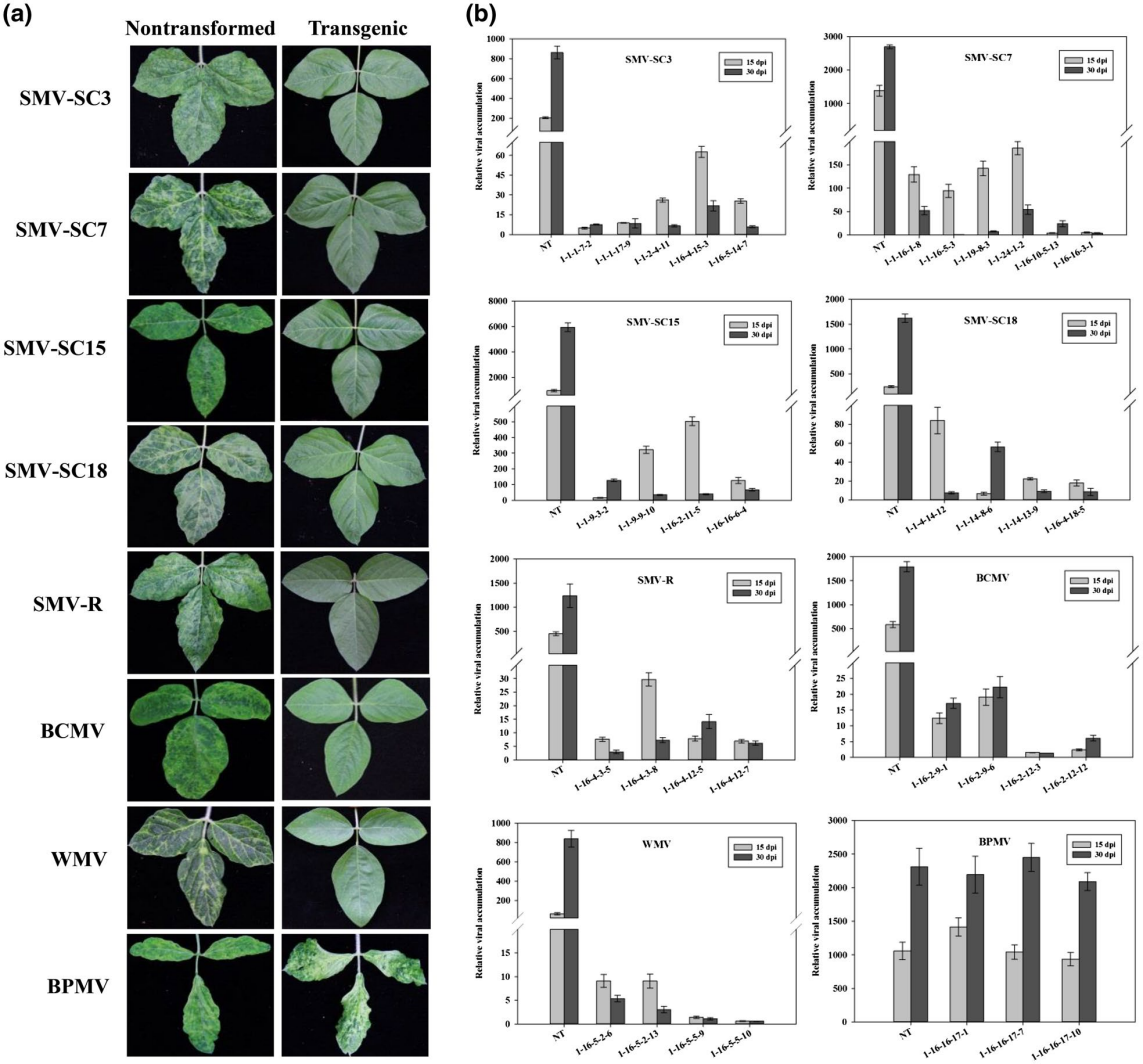
Broad‐spectrum resistance assessments in homozygous T_3_/T_4_ generations derived from T_0_ line 1. (a) Appearance of systemic symptoms on leaves of T_3_ soybean plants after challenge with different viruses. Virus‐inoculated nontransformed plants were used as controls. SMV, *soybean mosaic virus*; BCMV, bean common mosaic virus; WMV, watermelon mosaic virus; BPMV, bean pod mottle virus. (b) RT‐qPCR detection of systemic virus accumulation in leaves of T_4_ plants after challenge with different viruses. The *y* axes indicate virus transcript levels at 15 and 30 days post‐inoculation (dpi). The *x* axes indicate T_4_ and nontransformed (NT) plants. Results are representative of three independent experiments with error bars indicating *SD* (*n* = 3)

As shown in Table 2, highly resistant plants were the most numerous, and no susceptible plants were found in homozygous T_3_/T_4_ generations inoculated with the seven potyviruses (SMV, BCMV, and WMV). However, all T_3_/T_4_ plants were found to be susceptible to bean pod mottle virus (BPMV) (Table 2), indicating that eIF4E1‐mediated resistance was nonfunctional against BPMV, which may be due to its generic position (genus *Comovirus*; family *Secoviridae*). As shown in Figure 5a, compared with the leaves of nontransformed plants that exhibited a mosaic phenotype, the leaves of T_3_ plants were symptomless with normal morphology after being challenged with the seven potyviruses. However, BPMV‐inoculated T_3_ plants showed mosaic patterned and shrinking leaves, similar to those of the nontransformed plants (Figure 5a).

**Table 2:** Classification of responses of homozygous T_3_ and T_4_ soybean plants to different viruses

Virus	T_3_ generation^a^				T_4_ generation^a^			
	No. of plants evaluated	Highly resistant^b^	Mildly resistant^c^	Susceptible^d^	No. of plants evaluated	Highly resistant^b^	Mildly resistant^c^	Susceptible^d^
SMV‐SC3	42	42 (100.0%)	0 (0)	0 (0)	69	65 (94.2%)	4 (5.8%)	0 (0)
SMV‐SC7	40	36 (90.0%)	4 (10.0%)	0 (0)	61	48 (78.7%)	13 (21.3%)	0 (0)
SMV‐SC15	26	22 (84.6%)	4 (15.4%)	0 (0)	56	47 (83.9%)	9 (16.1%)	0 (0)
SMV‐SC18	20	17 (85.0%)	3 (15.0%)	0 (0)	41	31 (75.6%)	10 (24.4%)	0 (0)
SMV‐R	26	24 (92.3%)	2 (7.7%)	0 (0)	28	26 (92.9%)	2 (7.1%)	0 (0)
BCMV	12	10 (83.3%)	2 (16.7%)	0 (0)	12	12 (100.0%)	0 (0)	0 (0)
WMV	30	23 (76.7%)	7 (23.3%)	0 (0)	18	14 (77.8%)	4 (22.2%)	0 (0)
BPMV	15	0 (0)	0 (0)	15 (100.0%)	21	0 (0)	0 (0)	21 (100.0%)

Note

SMV, *soybean mosaic virus*; BCMV, bean common mosaic virus; WMV, watermelon mosaic virus; BPMV, bean pod mottle virus.

a Ten nontransformed plants were evaluated for each virus and they were all susceptible.

b Highly resistant plants with no visible viral symptoms.

c Mildly resistant plants with delayed appearance of viral symptoms or symptoms lighter than those of nontransformed controls.

d Susceptible plants with viral symptoms identical to those of nontransformed controls.

Based on RT‐qPCR analysis of the seven potyviruses, the virus content was found to increase dramatically in nontransformed plants, while it exhibited a decreasing tendency in most T_4_ plants, from 15 to 30 dpi (Figure 5b), and the varying virus transcript levels identified in different T_4_ plants were nearly background, being on average far less than those in nontransformed plants at both time points (Figure 5b). However, the virus content and variation were similar in BPMV‐inoculated nontransformed and T_4_ plants (Figure 5b), which was consistent with the results of resistance evaluation (Table 2 and Figure 5a). Additionally, analysis of virus accumulation in Kefeng 1 revealed an extremely low level in both inoculated and uninoculated leaves at different time points (Figure S2). Although the virus content in T_2_/T_4_ plants inoculated with SMV strain SC3 was far less than that in nontransformed plants (Figures 4b and 5b), it was still more than that of Kefeng 1 to a certain extent. We speculated that this resulted from the remaining low transcript levels of *eIF4E1* in transgenic plants (Figure 3a), which could sustain multiplication for a small amount of virus. DAS‐ELISA was performed with T_2_/T_3_ lines at 3 and 5 weeks post‐inoculation (wpi), and viral titers of T_2_/T_3_ lines separately challenged with the seven potyviruses were below 2.0, at both 3 and 5 wpi, demonstrating robust resistance to these viruses (Tables S5 and S6). However, consistent with the results of resistance evaluation (Table 2 and Figure 5a) and RT‐qPCR (Figure 5b), both nontransformed plants and transgenic lines were susceptible to BPMV (Tables S5 and S6).

In summary, these experiments provided evidence of the involvement of soybean *eIF4E1* in broad‐spectrum potyvirus resistance, suggesting that soybean *eIF4E1* is the susceptibility factor, not only for SMV, but also for BCMV and WMV.

## 3 DISCUSSION

The cap‐binding protein eIF4E participates in initiating mRNA translation and in controlling resistance/susceptibility to potyviruses. Subcellular localization showed that soybean eIF4E1 was simultaneously present in the nucleus and cytoplasm in *N. benthamiana* (Figure 2a), which is consistent with the recent findings of the localization of peanut eIF4E in the nucleus and cytoplasm in *Arabidopsis thaliana* (Xu *et al*., 2017). Physical interaction between eIF4E and VPg is a pivotal determinant of potyviral infectivity, along with a complex multistep process involved in virus translation, replication, intracellular trafficking, cell‐to‐cell movement, long‐distance migration, and suppression of host endogenous RNA silencing by disturbing siRNA and microRNA processing in the nucleus (Wang and Krishnaswamy, 2012; Sanfaçon, 2015). However, it is still not known if eIF4E–VPg interaction is the unique determinant of potyviral infectivity in a wide range of plant–potyvirus pairs (Mazier *et al*., 2011). As shown in Figure 2b,c, soybean eIF4E1 interacted with SMV VPg in the nucleus and with NIa‐Pro/NIb in the cytoplasm of *N. benthamiana*. In the peanut–peanut stripe virus pathosystem, eIF4E interacted with VPg in the nucleus and with HC‐Pro in the cytoplasm of *A. thaliana* (Xu *et al*., 2017). Thus, we speculated that potyviral proteins recognized by host eIF4E could be varied in different plant–virus pathosystems.

Considerable efforts have been made to exploit genes conferring resistance to diverse SMV strains in soybean. To date, four independent single‐dominant resistance loci (*Rsv1*, *Rsv3*, *Rsv4,* and *Rsv5*) and a series of *Rsc* loci conferring resistance to the U.S. and Chinese SMV strains have been fine‐mapped to soybean chromosomes 2, 6, 13, and 14 (MLG‐D1b, C2, F, and B2) (Hajimorad *et al*., 2018). Although *Rsv* and *Rsc* loci are located in close proximity to each other, the allelic relationship between them remains unclear, and none of these genes have been cloned thus far, therefore it is impossible to simply transform the resistance genes for generating transgenic SMV resistance (Liu *et al*., 2016; Hajimorad *et al*., 2018). In addition, the resistance spectrum of the *Rsv* and *Rsc* loci is limited or late‐susceptible, making it difficult to cultivate soybean varieties with multistrain SMV resistance through traditional breeding programmes, which is a labour‐intensive and time‐consuming process, and is always accompanied by the generation of undesirable traits (Gao *et al*., 2015a). Furthermore, strong selection pressure resulting from the extensive use of dominant genes is an important driving force for the frequent emergence of resistance‐breaking SMV strains/isolates (Steinlage *et al*., 2002; Gagarinova *et al*., 2008). In comparison with dominant resistance, recessive resistance is often broader and more durable because of its lower selective pressure on the viruses (Pyott *et al*., 2016; Gal‐On *et al*., 2017; Hajimorad *et al*., 2018).

High levels of transgenic SMV resistance have been successfully induced in soybean through RNAi (Zhang *et al*., 2011; Kim *et al*., 2013a, 2016; Gao *et al*., 2015a; Yang *et al*., 2017, 2018). However, RNA silencing in previous studies was confined to the viral genome, targeting *CP* (Kim *et al*., 2013a), *HC‐Pro* (Gao *et al*., 2015a; Kim *et al*., 2016), *NIb* (Zhang *et al*., 2011; Yang *et al*., 2017), and *P3* (Yang *et al*., 2018), and soybean endogenous genes have rarely been used for generating RNAi‐mediated SMV resistance. However, certain limitations exist in virus‐derived resistance via RNAi (Wang *et al*., 2013). Introduction of viral segments into plants might raise public concern and generate new viral variants through recombination between the introduced viral segments and other infecting viruses (Wang *et al*., 2013). Moreover, RNAi targeting viral genes may be hindered by the continuously evolving SMV population, possessing high variability along with error‐prone replication, mutation, and recombination; as a result, the specificity of the RNAi sequence would gradually be attenuated. Hence, silencing the soybean *eIF4E1*, as shown in the present study, can be an effective alternative for controlling SMV infections.

Functional redundancy has been observed between eIF4E and eIF(iso)4E in plant growth, and tobacco plants exhibited the semi‐dwarf phenotype only when *eIF4E* and *eIF(iso)4E* genes were simultaneously silenced (Combe *et al*., 2005). Previous studies using RNAi targeting eIF4E factors to generate viral resistance have shown differential developmental phenotypes in diverse crop species (Mazier *et al*., 2011; Rodríguez‐Hernández *et al*., 2012; Wang *et al*., 2013; Xu *et al*., 2017). Transgenic tomato lines silenced for eIF4E showed slightly impaired growth and fertility, while no obvious vegetative defects were observed in lines silenced for eIF(iso)4E; however, the F_1_ hybrid resulting from these two lines exhibited a pronounced semi‐dwarf phenotype, suggesting a cumulative effect of the silencing of *eIF4E* and *eIF(iso)4E* genes (Mazier *et al*., 2011). Eight transgenic melon lines silenced for eIF4E were obtained and self‐pollinated, of which only one T_0_ line produced abundant T_2_ seeds, as transgenesis often affected growth and fertility of the resulting plants (Rodríguez‐Hernández *et al*., 2012). Transgenic plum lines lacking either eIF4E or eIF(iso)4E did not show any phenotypic alterations, compared with the wild‐type plants, indicating a complementary effect of the two isoforms (Wang *et al*., 2013). Transgenic peanut plants silenced for eIF4E and/or eIF(iso)4E did not phenotypically differ from the control plants (Xu *et al*., 2017). In the present study, no apparent developmental defects were observed in the transgenic soybean plants silenced for eIF4E1, which might be due to the silencing effect not being thorough and the compensatory functions of other genes.

Previous research has confirmed that both eIF4E1 and eIF4E2 are involved in viral resistance in tomato (Mazier *et al*., 2011). In the present study, many mildly resistant (38.5%) and susceptible (27.7%) plants were identified in the T_1_ generation (Table 1), implying that most T_0_ lines did not trigger much SMV resistance, although they exhibited a strong reduction in *eIF4E1* transcript accumulation (Figure 3a). Interestingly, only one (T_0_ line 1) of the 18 T_0_ lines showed significant resistance and all its T_1_ progenies were highly resistant to SMV (Table 1). Hence, we speculated that in T_0_ line 1, soybean *eIF4E2* was also silenced, which enhanced the viral resistance. To verify this hypothesis, 24 T_5_ plants derived from T_0_ line 1 were randomly selected for RT‐qPCR analysis of the *eIF4E1* and *eIF4E2* (primer 5 in Table S1) transcript levels. As shown in Figure S3, a significant decrease in transcript accumulation was observed in T_5_ plants, not only in *eIF4E1* (more than 90% decrease), but also in *eIF4E2* (60–90% decrease), when compared with nontransformed plants. This demonstrated that the enhanced viral resistance in the T_0_ line 1 could be attributed to the simultaneous silencing of soybean *eIF4E1* and *eIF4E2*, which is consistent with the fact that both *eFI4E1* and *eIF4E2* have to be down‐regulated for viral resistance in tomato (Mazier *et al*., 2011). We can therefore conclude that soybean eIF4E1 and eIF4E2 play overlapping or redundant roles in the virus multiplication cycle.

SMV, BCMV, and WMV can infect soybean crops, resulting in yield reductions, and mixed infections and synergistic interactions are common among these viruses in Chinese field‐grown soybean plants (Zhou *et al*., 2014; Yang *et al*., 2017, 2018). Furthermore, genetic exchanges among SMV, BCMV, and WMV occur frequently, and recombinant SMV variants have been reported prevalent in Chinese soybean fields, presenting a complicated and severe challenge to soybean farming in China (Yang *et al*., 2011, 2014; Zhou *et al*., 2015; Chen *et al*., 2017; Jiang *et al*., 2017). Hence, it is imperative to confer soybean plants with resistance, not only against SMV, but also against BCMV and WMV. In this study, a high level of broad‐spectrum resistance to five SMV strains (SC3/7/15/18 and SMV‐R), BCMV, and WMV was developed in transgenic soybean (Tables 1 and 2, Figures 4 and 5, and Tables [Link], [Link], [Link]). Our results suggest that eIF4E‐mediated resistance to potyviruses, based on RNAi, is effective and broad‐spectrum, providing an efficient strategy for combatting viral pathogens in soybean.

## 4 EXPERIMENTAL PROCEDURES

### 4.1 Expression analysis of soybean *eIF4E1* using RT‐qPCR

Spatiotemporal expression profiles of *eIF4E1* were explored in soybean cultivars Tianlong 1 (SMV susceptible) and Kefeng 1 (SMV resistant), through RT‐qPCR. To detect the temporal responses of *eIF4E1* to SMV infection, both Tianlong 1 and Kefeng 1 were mechanically inoculated with SMV strain SC3 and 0.01 M phosphate‐buffered saline (PBS), and samples were collected independently from the inoculated leaves at different time points (0, 1, 2, 4, 8, 12, 24, 48, and 72 hpi). Inoculation was performed as previously described (Li *et al*., 2010), and the relative expression levels were calibrated using mock‐inoculated (inoculated with PBS) controls. To determine the spatial expression patterns of *eIF4E1*, samples were collected from various healthy soybean tissues, including roots, stems, leaves, flowers, immature pods, and mature seeds, from Tianlong 1 and Kefeng 1. Roots, stems, and leaves were collected at the V2 stage, flowers were collected at the R2 stage, and immature pods were collected at the R5 stage. All samples were stored at −80 °C until RT‐qPCR analysis.

Gene‐specific primers for RT‐qPCR were designed targeting soybean *eIF4E1* (primer 3 in Table S1), using Primer Premier 5.0 software, and the gene *Tubulin* (accession no. AY907703; primer 6 in Table S1) was used as an internal reference control. Total RNA extractions and first‐strand cDNA syntheses were performed using an RNA Simple Total RNA Kit (Tiangen) and PrimeScript RT Master Mix (Takara), respectively, according to the manufacturer's instructions. RT‐qPCR was performed in a 20‐μL final volume, containing 2 μL of template cDNA (approximately 50 ng), 0.4 μL of each primer (10 μM), 10 μL of 2 × SYBR Premix Ex Taq (Takara), and 7.2 μL of sterilized double‐distilled water. Thermal conditions were set as follows: 95 °C for 30 s; followed by 40 cycles at 95 °C for 5 s, 55 °C for 30 s, and 72 °C for 30 s. Reactions were analysed in triplicate, in 96‐well plates, on a LightCycler 480 II (Roche). Transcript levels were quantified using the relative quantification (2^‐ΔΔCt^) method (Livak and Schmittgen, 2001) and data were compared with internal controls.

### 4.2 Subcellular localization

The 711‐bp full‐length coding sequence of *eIF4E1* (primer 1 in Table S1) without its stop codon was amplified from Tianlong 1 by RT‐PCR using KOD FX (Toyobo). According to the manufacturer's manual for the Gateway system (Invitrogen), *eIF4E1* was successively ligated to the entry vector pDONR/Zeo and then to the destination vector pGWB6 using BP and LR clonases. The recombinant plasmid expressing the eIF4E1‐GFP fusion protein was introduced into *Agrobacterium tumefaciens* EHA105 via electroporation. Agrobacterial cultures were grown overnight in a shaker incubator at 200 rpm at 28 °C, and *A. tumefaciens* cells were pelleted by centrifugation and subsequently resuspended in infiltration buffer (10 mM MgCl_2_, 10 mM MES, 150 μM acetosyringone, pH 5.6). The *A. tumefaciens* cell suspension was adjusted to an optical density of 0.6–0.8 at 600 nm (OD_600_) and agroinfiltrated into leaves of 4‐week‐old *N. benthamiana* using a 1‐mL syringe without the needle. The GFP signal was visualized under a spectral confocal laser scanning microscope (Carl Zeiss).

### 4.3 Y2H and BiFC assays

Y2H screening was performed using the Matchmaker DUAL membrane system (Dualsystems Biotech) according to the manufacturer's protocols. The *eIF4E1* of Tianlong 1 and 11 genes of SMV strain SC3 (primers 8–18 in Table S1) were amplified by RT‐PCR using KOD FX. The *eIF4E1* was digested with *Sfi*I and then ligated to the bait vector pBT3‐STE, and 11 SMV genes were individually cloned into the prey vector pPR3 using the Gateway system. The correct bait and prey vectors, verified by sequencing, were co‐transformed into yeast cells (*Saccharomyces cerevisiae* NMY51). Selective quadruple dropout *SD*/−Leu/−Trp/−Ade/−His/+X‐α‐Gal media were used to detect any protein–protein interactions, and blue colonies were considered positive. Yeast containing pBT3‐STE + pPR3, pBT3‐STE‐eIF4E1 + pPR3, or pBT3‐STE + pPR3‐SMV served as negative controls. Yeast cells co‐transformed with pPR3‐P3N‐PIPO + pBT3‐STE‐GOS12 were used as positive control (Song *et al*., 2016).

For the BiFC assay, yellow fluorescent protein (YFP) was reconstituted by co‐expressing the corresponding protein pairs in *N. benthamiana* leaf cells via agroinfiltration. The *eIF4E1* of Tianlong 1 and three genes of SMV strain SC3 (i.e., *VPg*, *NIa‐Pro*, and *NIb*) were introduced into the Gateway vectors pEarleyGate202‐YN and pEarleyGate201‐YC, respectively, and then individually electrotransformed into *A. tumefaciens* EHA105. A mixture of two agrobacterial cultures was resuspended in infiltration buffer (OD_600_ = 0.6–0.8) and agroinfiltrated into 4‐week‐old *N. benthamiana* leaves. Interactions between YN and YC, YN and SMV‐YC, and eIF4E1‐YN and YC were used as negative controls. YFP expression was observed under a confocal microscope.

### 4.4 Western blot analysis

The expression of fusion proteins in subcellular localization (Figure S4a) and BiFC (Figure S4b,c) was verified by western blot analysis. Total proteins were extracted from *N. benthamiana* by grinding frozen leaf tissues (1 g) in buffer containing 50 mM Tris–HCl (pH 7.5), 10% glycerol, 150 mM NaCl, 10 mM MgCl_2_, 5 mM EDTA, 5 mM DTT, and 1 × protease inhibitor cocktail (Sigma‐Aldrich). The homogenate was centrifuged at 10,000 g, followed by a second centrifugation at 125,000 g. Proteins (40 μg per lane) were separated by 12% SDS‐PAGE at 100 V for 1–2 hr, transferred to nitrocellulose membrane (GE Water and Process Technologies), and detected using protein/tag‐specific antibodies (Figure S4).

### 4.5 Vector construction, soybean transformation, and confirmation of transgene‐positive plants

The 348‐bp RNAi fragment *eIF4E1i* (primer 2 in Table S1) was amplified from the *eIF4E1* coding sequence (nucleotide sites 267–614) of Tianlong 1 by RT‐PCR and recombined into the vector pB7GWIWG2(II) using the Gateway system. The resulting recombinant construct (Figure S1) contained the phosphinothricin acetyltransferase (*bar*) gene conferring resistance to the herbicide phosphinothricin and was introduced into *A. tumefaciens* EHA105. Tianlong 1 was used in the cotyledonary node‐*Agrobacterium*‐mediated transformation system and putative transformants were simultaneously verified by leaf‐painting, PCR, and LibertyLink strip. Soybean transformation and confirmation of transgene‐positive plants were performed as previously described (Gao *et al*., 2015a).

### 4.6 Southern blot hybridization analysis

Total genomic DNA (c.30 μg) was digested completely with the *Hin*dIII restriction endonuclease (Thermo), which recognizes a unique site within the T‐DNA region (Figure S1). Digested DNA was separated on 0.8% agarose gel and transferred to Hybond‐N^+^ nylon membrane (Amersham). A PCR‐generated *bar* gene fragment (primer 7 in Table S1) labelled with digoxigenin (DIG)‐High Prime (Roche) was used as a probe (Figure S1). Prehybridization, hybridization, membrane washing, and signal detection were carried out using DIG‐High Prime DNA Labeling and Detection Starter Kit II (Roche), according to the manufacturer's protocols.

### 4.7 Virus inoculation and resistance assessment

Five SMV strains (SC3/7/15/18 and SMV‐R), BCMV, WMV, and BPMV were individually maintained in soybean cultivar Nannong 1138–2 (highly susceptible host) and used for resistance evaluation. Mechanical inoculation was carried out in an insect‐proof greenhouse as previously described (Li *et al*., 2010), and plants were regularly sprayed with pesticides to prevent cross‐infection via aphids.

T_1_/T_2_ generations were evaluated for resistance to SMV strain SC3, and T_3_/T_4_ generations were assessed for broad‐spectrum resistance against SC3, SC7, SC15, SC18, SMV‐R, BCMV, WMV, and BPMV. Viral symptoms (including no symptoms, mosaic pattern, and necrosis) were visually observed and noted at 1‐week intervals until the R1 stage in the inoculated plants. Responses of transgenic plants were classified as follows: (a) highly resistant plants with no visible viral symptoms, (b) mildly resistant plants with delayed appearance of viral symptoms or symptoms lighter than those of nontransformed controls, and (c) susceptible plants with viral symptoms identical to those of nontransformed controls.

### 4.8 Molecular detection of virus accumulation in transgenic soybeans

At the transcriptional level, virus accumulation in T_2_/T_4_ generations was detected by RT‐qPCR analysis of the viral *CP* genes (primers 19–22 in Table S1), and the gene *Tubulin* was used as an internal reference control. Leaf samples were independently collected from the uninoculated leaves of inoculated transgenic and nontransformed plants at 15 and 30 dpi. In addition, virus accumulation was detected in Kefeng 1, by RT‐qPCR, after challenge with SMV strain SC3. Leaf samples were independently collected at different time points from the inoculated (0, 12, 24, and 72 hpi, and 5 dpi) and uninoculated leaves of inoculated plants (7, 10, and 15 dpi). Methods for total RNA extractions, cDNA syntheses, and RT‐qPCR analyses have been described in previous sections.

At the translational level, systemic virus content in uninoculated leaves of T_2_–T_4_ generations was assessed by DAS‐ELISA. Kits complete with anti‐SMV, anti‐BCMV, anti‐WMV, and anti‐BPMV antibodies (AC Diagnostics) were used, following the manufacturer's instructions. Forty‐two T_2_ plants, 12 T_2_ lines, and 25 T_3_ lines were selected for evaluation, and virus‐inoculated and mock‐inoculated nontransformed plants were used as positive and negative controls, respectively. Five T_3_/T_4_ plants were randomly selected from each of the tested T_2_/T_3_ lines, and the average reading of the five plants represented the value for the line. T_2_ plants and T_2_/T_3_ lines with relative values greater than 2.0 were considered susceptible to the virus.

## CONFLICT OF INTERESTS

The authors have no conflicts of interest.

## Supporting information


**Text S1** Sequences of soybean eIF4E1 and eIF(iso)4E1 from Nannong 1138‐2 and five mutant cultivars

**Fig S1** Schematic representation of the T‐DNA region of the recombinant plasmid pB7GWIWG2(II)‐*eIF4E1i* used for soybean transformation. LB/RB, left/right border; *bar*, phosphinothricin acetyltransferase gene; P35S/T35S, CaMV 35S promoter/terminator; *CmR*, chloramphenicol resistance gene. *Hin*dIII recognizes a single restriction enzyme site within pB7GWIWG2(II)‐*eIF4E1i*. A *bar* probe specific to the *bar *gene region was used for Southern blot hybridization analysis

**Fig S2** RT‐qPCR detection of virus accumulation in Kefeng 1 after challenge with *soybean mosaic virus* (SMV) strain SC3. The *y* axis indicates SMV transcript levels. The *x* axis indicates leaf samples collected from inoculated or systemic leaves at different time points. hpi, hours post‐inoculation; dpi, days post‐inoculation. Results are representative of three independent experiments, with error bars indicating *SD* (*n* = 3)

**Fig S3** RT‐qPCR detection of the relative expression levels of soybean *eIF4E1* and *eIF4E2* in T_5_ plants derived from T_0 _line 1. The *y* axis indicates transcript levels of *eIF4E1* and *eIF4E2*. The *x* axis indicates T_5_ and nontransformed (NT) plants. Results are representative of three independent experiments, with error bars indicating *SD* (*n* = 3)

**Fig S4** Western blot analysis confirming the expression of fusion proteins in *Nicotiana benthamiana* for subcellular localization and bimolecular fluorescence complementation (BiFC) assay. (a) Fusion proteins for subcellular localization detected using green fluorescent protein (GFP) antibody. (b) Fusion proteins for BiFC detected using FLAG‐Tag antibody. (c) Fusion proteins for BiFC detected using HA‐Tag antibody. Positions of protein mobility markers in kilodaltons (kDa) are indicated on the left

**Table S1** Sequences of primer pairs used in this study

**Table S2** Efficiency of cotyledonary node‐*Agrobacterium*‐mediated soybean transformation. All positive plants were confirmed using leaf‐painting, PCR and LibertyLink strip. Transformation efficiency = (no. of positive T_0_ plants / no. of infected explants) × 100. Data was expressed as mean ± *SD*

**Table S3** Investigation of seed coat mottling in T_1_–T_4_ lines after challenge with *soybean mosaic virus* (SMV) strain SC3. NT, nontransformed plant. Mottling rate = (total no. of mottled seeds / total no. of seeds) × 100

**Table S4** DAS‐ELISA analysis of T_2_ plants inoculated with *soybean mosaic virus* (SMV) strain SC3. +, positive for SMV; ‐, negative for SMV; NT, nontransformed plant. OD_405 _value of each sample was calculated by averaging the three readings of the plate. OD_405_ value of negative control (mock inoculation) was calculated by averaging the three readings of the plate, which was 0.183

**Table S5** DAS‐ELISA analysis of T_2_ lines inoculated with different viruses. SMV, soybean mosaic virus; BCMV, bean common mosaic virus; WMV, watermelon mosaic virus; BPMV, bean pod mottle virus; NT, nontransformed plant; wpi, weeks post‐inoculation; +, positive for virus; −, negative for virus. OD_405_ value of each T_2_ line was calculated by averaging the values of five T_3 _plants randomly selected from the line. OD_405_ value of each positive control was calculated by averaging the values of three virus‐inoculated NT plants, and OD_405_ value of each negative control was calculated by averaging the values of three mock‐inoculated NT plants

**Table S6** DAS‐ELISA analysis of T_3_ lines inoculated with different viruses. SMV, soybean mosaic virus; BCMV, bean common mosaic virus; WMV, watermelon mosaic virus; BPMV, bean pod mottle virus; NT, nontransformed plant; wpi, weeks post‐inoculation; +, positive for virus; −, negative for virus. OD_405_ value of each T_3_ line was calculated by averaging the values of five T_4 _plants randomly selected from the line. OD_405_ value of each positive control was calculated by averaging the values of three virus‐inoculated NT plants, and OD_405_ value of each negative control was calculated by averaging the values of three mock‐inoculated NT plants

**Table S7** The 208 soybean cultivars used for SMV resistance assessment. SMV, soybean mosaic virus_._ Seventeen soybean cultivars identified as SMV‐resistant are highlighted in bold

**Table S8** Sequencing analysis of *eIF4E1* from the 17 SMV‐resistant soybean cultivars and protein–protein interactions between mutated eIF4E1s and SMV VPg via Y2H. D, aspartic acid; H, histidine; K, lysine; N, asparagine; R, arginine; SMV, soybean mosaic virus; VPg, viral genome‐linked protein; Y2H, yeast two‐hybrid; +, interaction with SMV VPg; −, no interaction with SMV VPg. All mutations were compared with the soybean cultivar Nannong 1138‐2 (highly susceptible host)

## Data Availability

The data that support the findings of this study are available from the corresponding author upon reasonable request.
