# Supplementary material for: Soybean RNA interference lines silenced for eIF4E show broad potyvirus resistance
Source: Mol Plant Pathol. 2019 Dec 20;21(3):303–17. doi: 10.1111/mpp.12897 (PMC7036369; doi:10.1111/mpp.12897)
Supplement: Supplementary file 1 — Text S1 Sequences of soybean eIF4E1 and eIF(iso)4E1 from Nannong 1138‐2 and five mutant cultivars [file MPP-21-303-s001.docx]

**Text S1** Sequences of soybean eIF4E1 and eIF(iso)4E1 from Nannong 1138-2 and five mutant cultivars.

1. **Nannong 1138-2**

**eIF4E1 (nucleotide sequence, accession No. MN369710)**

ATGGTTGTAGAAGATACCCAAAAGTCTGTCATCACGGAGGACCAATACCCTAGCAGGGTCGTCAGCGACAACAACAACGACGACGACGACGATGATCTCGAAGAGGGTGAGATCCCCGTCGACGGCGAAGATAGCGGCGCCACCGCCACAACGAAGCCTCCGGCCGCCCTCGCCCGCAACCCCCACCCTCTGGAGAATTCCTGGACCTTCTGGTTCGACAACCCTTCCTCCAAGTCCAAACAAGCCGCATGGGGCAGCTCCATCCGACCCATCTACACTTTCGCCACCGTTGAAGAGTTTTGGAGCATTTACAATAACATTCACCACCCGAGCAAGTTGGGTTTGGGGGCGGACTTTCACTGCTTCAAGCACAAGATTGAGCCAAAGTGGGAGGACCCTATCTGCGCCAATGGGGGAAAGTGGACTATGACCTTCCCAAGGGGGAAATCTGATACCAGTTGGTTGTATACGTTGTTGGCGATGATTGGAGAACAGTTTGATCACGGAGATGAAATATGTGGAGCTGTTGTGAATGTCAGAAGTAGGCAGGATAAAATTGCTATTTGGACTAAGAACGCTTCAAATGAAGCTGCTCAGGTGAGCATTGGAAAGCAGTGGAAGGAGTTTCTTGATTACAATGACACAATTGGCTTTATATTTCATGAGGATGCAAAGAAGCTGGACAGAGGTGCTAAAAATAAATACGTTGTATGA

**eIF4E1 (amino acid sequence)**

MVVEDTQKSVITEDQYPSRVVSDNNNDDDDDDLEEGEIPVDGEDSGATATTKPPAALARNPHPLENSWTFWFDNPSSKSKQAAWGSSIRPIYTFATVEEFWSIYNNIHHPSKLGLGADFHCFKHKIEPKWEDPICANGGKWTMTFPRGKSDTSWLYTLLAMIGEQFDHGDEICGAVVNVRSRQDKIAIWTKNASNEAAQVSIGKQWKEFLDYNDTIGFIFHEDAKKLDRGAKNKYVV

**eIF(iso)4E1 (nucleotide sequence, accession No. MN369709)**

ATGGCAACAAGCGAAGAAGTGGTTGCGGCGGCGCCGGAGGCAGCCGCGCCGGAGGCAGGGTTGAAGCACAAACTGGAGAGAAAATGGACGTTTTGGTGCGACAACCAATCCAAACCCAAGCAAGGCGCTGCTTGGGGAACCTCTCTTCGCAAGGTCTACACCTTCGACACCGTTGAAGAGTTCTGGTGTTTGTATGATCAGGTATTCAAGCCCAGCAAGTTGCAAATCAATGCCGATTTTCACTTGTTCAAGACTGGGATTGACCCTAAATGGGAAGATCCAGAGTGCGCCAATGGAGGAAAGTGGTCTATCACCAGCAACAGCGGCAGGAAGGCTAACCTTGATAACATGTGGCTTGAAACTATGATGGCTTTGATTGGGGAACAATTTGAGGATGCTGAGGACATATGTGGTGTGGTTGCTAGTGTGCGCCAGTGGCAGGACAAACTTTCGTTGTGGACAAAGACAGCAGCAAATGAAGCTGCCCAGATGAGCATTGGAAGGAAGTGGAAGGAAATCATTGATGTTAACGACAAGATAACATACAACTTTCATGATGATTCTAGAAGCAAAGGAGCAACAAAGGGTCGGTACACCGTATAA

**eIF(iso)4E1 (amino acid sequence)**

MATSEEVVAAAPEAAAPEAGLKHKLERKWTFWCDNQSKPKQGAAWGTSLRKVYTFDTVEEFWCLYDQVFKPSKLQINADFHLFKTGIDPKWEDPECANGGKWSITSNSGRKANLDNMWLETMMALIGEQFEDAEDICGVVASVRQWQDKLSLWTKTAANEAAQMSIGRKWKEIIDVNDKITYNFHDDSRSKGATKGRYTV

1. **Zhongzuo 02-760**

**eIF4E1 (nucleotide sequence, accession No.** **MN369711)**

ATGGTTGTAGAAGATACCCAAAAGTCAGTCATCACGGAGGACCAATACCCTAGCAGGGTCGTCAGCGACAACAACAACGACGACGACGACGATGATCTCGAAGAGGGTGAGATCCCCGTCGACGGCGAAGATAGCGGCGCCACCGCCACAACGAAGCCTCCGGCCGCCCTCGCCCGAAACCCCCACCCTCTGGAGAATTCCTGGACCTTCTGGTTCGACAACCCTTCCTCCAAGTCCAAACAAGCCGCATGGGGCAGCTCCATCCGACCCATCTACACTTTCGCCACCGTTGAAGAGTTTTGGAGCATTTACAATAACATTCACCACCCGAGCAAGTTGGGTTTGGGGGCGGACTTTCACTGCTTCAAGCACAAGATTGAGCCAAAGTGGGAGGACCCTATCTGCGCCAATGGGGGAAAGTGGACTATGACCTTCCCAAGGGGAAAATCTGATACCAGTTGGTTGTATACGTTGTTGGCGATGATTGGAGAACAGTTTGATCACGGAAATGAAATATGTGGAGCTGTTGTGAATGTCAGAAGTAGGCAGGATAAAATTGCTATTTGGACTAAAAACGCTTCAAATGAAGCTGCTCAGGTGAGCATTGGAAAGCAGTGGAAGGAGTTTCTTGATTACAATGACACAATTGGCTTTATATTTCATGAGGATGCAAAGAAGCTGGACAGAGGTGCTAAAAATAAATACGTTGTATGA

**eIF4E1 (amino acid sequence)**

MVVEDTQKSVITEDQYPSRVVSDNNNDDDDDDLEEGEIPVDGEDSGATATTKPPAALARNPHPLENSWTFWFDNPSSKSKQAAWGSSIRPIYTFATVEEFWSIYNNIHHPSKLGLGADFHCFKHKIEPKWEDPICANGGKWTMTFPRGKSDTSWLYTLLAMIGEQFDHGNEICGAVVNVRSRQDKIAIWTKNASNEAAQVSIGKQWKEFLDYNDTIGFIFHEDAKKLDRGAKNKYVV

1. **Zhongzuo 06-06**

**eIF4E1 (nucleotide sequence, accession No.** **MN369712)**

ATGGTTGTAGAAGATACCCAAAAGTCAGTCATCACGGAGGACCAATACCCTAGCAGGGTCGTCAGCGACAACAACAACGACGACGACGACGATGATCTCGAAGAGGGTGAGATCCCCGTCGACGGCGAAGATAGCGGCGCCACCGCCACAACGAAGCCTCCGGCCGCCCTCGCCCGAAACCCCCACCCTCTGGAGAATTCCTGGACCTTCTGGTTCGACAACCCTTCCTCCAAGTCCAAACAAGCCGCATGGGGCAGCTCCATCCGACCCATCTACACTTTCGCCACCGTTGAAGAGTTTTGGAGCATTTACAATAACATTCACCACCCGAGCAAGTTGGGTTTGGGGGCGGACTTTCACTGCTTCAAGCACAAGATTGAGCCAAAGTGGGAGGACCCTATCTGCGCCAATGGGGGAAAGTGGACTATGACCTTCCCAAGGGGAAAATCTGATACCAGTTGGTTGTATACGTTGTTGGCGATGATTGGAGAACAGTTTGATCACGGAAATGAAATATGTGGAGCTGTTGTGAATGTCAGAAGTAGGCAGGATAAAATTGCTATTTGGACTAAAAACGCTTCAAATGAAGCTGCTCAGGTGAGCATTGGAAAGCAGTGGAAGGAGTTTCTTGATTACAATGACACAATTGGCTTTATATTTCATGAGGATGCAAAGAAGCTGGACAGAGGTGCTAAAAATAAATACGTTGTATGA

**eIF4E1 (amino acid sequence)**

MVVEDTQKSVITEDQYPSRVVSDNNNDDDDDDLEEGEIPVDGEDSGATATTKPPAALARNPHPLENSWTFWFDNPSSKSKQAAWGSSIRPIYTFATVEEFWSIYNNIHHPSKLGLGADFHCFKHKIEPKWEDPICANGGKWTMTFPRGKSDTSWLYTLLAMIGEQFDHGNEICGAVVNVRSRQDKIAIWTKNASNEAAQVSIGKQWKEFLDYNDTIGFIFHEDAKKLDRGAKNKYVV

1. **Zhongzuo J8035**

**eIF4E1 (nucleotide sequence, accession No.** **MN369713)**

ATGGTTGTAGAAGATACCCAAAAGTCTGTCATCACGGAGGACCAATACCCTAGCAGGGTCGTCAGCGACAACAACAACGACGACGACGACGATGATCTCGAAGAGGGTGAGATCCCCGTCGACGGCGAAGATAGCGGCGCCACCGCCACAACGAAGCCTCCGGCCGCCCTCGCCCGCAACCCCCACCCTCTGGAGAATTCCTGGACCTTCTGGTTCGACAACCCTTCCTCCAAGTCCAAACAAGCCGCATGGGGCAGCTCCATCCGACCCATCTACACTTTCGCCACCGTTGAAGAGTTTTGGAGCATTTACAATAACATTCACCACCCGAGCAAGTTGGGTTTGGGGGCGGACTTTCACTGCTTCAAGCACAAGATTGAGCCAAAGTGGGAGGACCCTATCTGCGCCAATGGGGGAAAGTGGACTATGACCTTCCCAAGGGGGAAATCTGATACCAGTTGGTTGTATACGTTGTTGGCGATGATTGGAGAACAGTTTGATCACGGAGATGAAATATGTGGAGCTGTTGTGAATGTCAAAAGTAGGCAGGATAAAATTGCTATTTGGACTAAAAACGCTTCAAATGAAGCTGCTCAGGTGAGCATTGGAAAGCAGTGGAAGGAGTTTCTTGATTACAATGACACAATTGGCTTTATATTTCATGAGGATGCAAAGAAGCTGGACAGAGGTGCTAAAAATAAATACGTTGTATGA

**eIF4E1 (amino acid sequence)**

MVVEDTQKSVITEDQYPSRVVSDNNNDDDDDDLEEGEIPVDGEDSGATATTKPPAALARNPHPLENSWTFWFDNPSSKSKQAAWGSSIRPIYTFATVEEFWSIYNNIHHPSKLGLGADFHCFKHKIEPKWEDPICANGGKWTMTFPRGKSDTSWLYTLLAMIGEQFDHGDEICGAVVNVKSRQDKIAIWTKNASNEAAQVSIGKQWKEFLDYNDTIGFIFHEDAKKLDRGAKNKYVV

1. **BQ15**

**eIF4E1 (nucleotide sequence, accession No. MN369714)**

ATGGTTGTAGAAGATACCCAAAAGTCTGTCATCACGGAGGACCAATACCCTAGCAGGGTCGTCAGCGACAACAACAACGACGACGACGACCATGATCTCGAAGAGGGTGAGATCCCCGTCGACGGCGAAGATAGCGGCGCCACCGCCACAACGAAGCCTCCGGCCGCCCTCGCCCGCAACCCCCACCCTCTGGAGAATTCCTGGACCTTCTGGTTCGACAACCCTTCCTCCAAGTCCAAACAAGCCGCATGGGGCAGCTCCATCCGACCCATCTACACTTTCGCCACCGTTGAAGAGTTTTGGAGCATTTACAATAACATTCACCACCCGAGCAAGTTGGGTTTGGGGGCGGACTTTCACTGCTTCAAGCACAAGATTGAGCCAAAGTGGGAGGACCCTATTTGCGCCAATGGGGGAAAGTGGACTATGACCTTCCCAAGGGGGAAATCTGATACCAGTTGGTTGTATACGTTGTTGGCGATGATTGGAGAACAGTTTGATCACGGAGATGAAATATGTGGAGCTGTTGTGAATGTCAGAAGTAGGCAGGATAAAATTGCTATTTGGACTAAGAACGCTTCAAATGAAGCTGCTCAGGTGAGCATTGGAAAGCAGTGGAAGGAGTTTCTTGATTACAATGACACAATTGGCTTTATATTTCATGAGGATGCAAAGAAGCTGGACAGAGGTGCTAAAAATAAATACGTTGTATGA

**eIF4E1 (amino acid sequence)**

MVVEDTQKSVITEDQYPSRVVSDNNNDDDDHDLEEGEIPVDGEDSGATATTKPPAALARNPHPLENSWTFWFDNPSSKSKQAAWGSSIRPIYTFATVEEFWSIYNNIHHPSKLGLGADFHCFKHKIEPKWEDPICANGGKWTMTFPRGKSDTSWLYTLLAMIGEQFDHGDEICGAVVNVRSRQDKIAIWTKNASNEAAQVSIGKQWKEFLDYNDTIGFIFHEDAKKLDRGAKNKYVV

1. **Liao 04M05-3**

**eIF4E1 (nucleotide sequence, accession No.** **MN369715)**

ATGGTTGTAGAAGATACCCAAAAGTCAGTCATCACGGAGGACCAATACCCTAGCAGGGTCGTCAGCGACAACAACAACGACGACGACGACGATGATCTCGAAGAGGGTGAGATCCCCGTCGACGGCGAAGATAGCGGCGCCACCGCCACAACGAAGCCTCCGGCCGCCCTCGCCCGAAACCCCCACCCTCTGGAGAATTCCTGGACCTTCTGGTTCGACAACCCTTCCTCCAAGTCCAAACAAGCCGCATGGGGCAGCTCCATCCGACCCATCTACACTTTCGCCACCGTTGAAGAGTTTTGGAGCATTTACAATAACATTCACCACCCGAGCAAGTTGGGTTTGGGGGCGGACTTTCACTGCTTCAAGCACAAGATTGAGCCAAAGTGGGAGGACCCTATCTGCGCCAATGGGGGAAAGTGGACTATGACCTTCCCAAGGGGAAAATCTGATACCAGTTGGTTGTATACGTTGTTGGCGATGATTGGAGAACAGTTTGATCACGGAGATGAAATATGTGGAGCTGTTGTGAATGTCAAAAGTAGGCAGGATAAAATTGCTATTTGGACTAAGAACGCTTCAAATGAAGCTGCTCAGGTGAGCATTGGAAAGCAGTGGAAGGAGTTTCTTGATTACAATGACACAATTGGCTTTATATTTCATGAGGATGCAAAGAAGCTGGACAGAGGTGCTAAAAATAAATACGTTGTATGA

**eIF4E1 (amino acid sequence)**

MVVEDTQKSVITEDQYPSRVVSDNNNDDDDDDLEEGEIPVDGEDSGATATTKPPAALARNPHPLENSWTFWFDNPSSKSKQAAWGSSIRPIYTFATVEEFWSIYNNIHHPSKLGLGADFHCFKHKIEPKWEDPICANGGKWTMTFPRGKSDTSWLYTLLAMIGEQFDHGDEICGAVVNVKSRQDKIAIWTKNASNEAAQVSIGKQWKEFLDYNDTIGFIFHEDAKKLDRGAKNKYVV
