# Supplementary material for: Soybean RNA interference lines silenced for eIF4E show broad potyvirus resistance
Source: Mol Plant Pathol. 2019 Dec 20;21(3):303–17. doi: 10.1111/mpp.12897 (PMC7036369; doi:10.1111/mpp.12897)
Supplement: Supplementary file 2 — Fig S1 Schematic representation of the T‐DNA region of the recombinant plasmid pB7GWIWG2(II)‐eIF4E1i used for soybean transformation. LB/RB, left/right border; bar, phosphinothricin acetyltransferase gene; P35S/T35S, CaMV 35S promoter/terminator; CmR, chloramphenicol resistance gene. HindIII recognizes a single restriction enzyme site within pB7GWIWG2(II)‐eIF4E1i. A bar probe specific to the bar gene region was used for Southern blot hybridization analysis [file MPP-21-303-s002.docx]

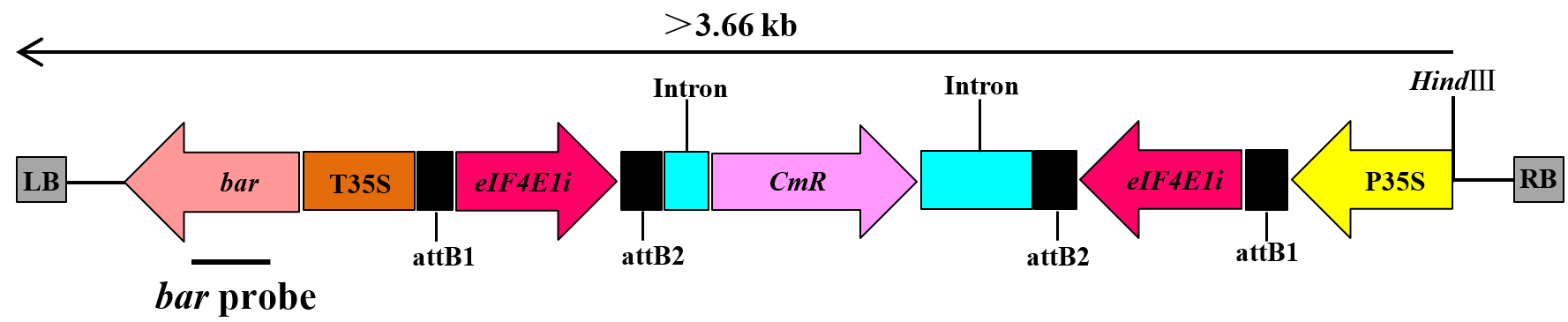


**Fig. S1** Schematic representation of the T-DNA region of the recombinant plasmid pB7GWIWG2(II)-*eIF4E1i* used for soybean transformation. LB/RB, left/right border; *bar*, phosphinothricin acetyltransferase gene; P35S/T35S, CaMV 35S promoter/terminator; *CmR*, chloramphenicol resistance gene. *Hind*III recognizes a single restriction enzyme site within pB7GWIWG2(II)-*eIF4E1i*. A *bar* probe specific to the *bar* gene region was used for Southern blot hybridization analysis.
