# Supplementary material for: Soybean RNA interference lines silenced for eIF4E show broad potyvirus resistance
Source: Mol Plant Pathol. 2019 Dec 20;21(3):303–17. doi: 10.1111/mpp.12897 (PMC7036369; doi:10.1111/mpp.12897)
Supplement: Supplementary file 3 — Fig S2 RT‐qPCR detection of virus accumulation in Kefeng 1 after challenge with soybean mosaic virus (SMV) strain SC3. The y axis indicates SMV transcript levels. The x axis indicates leaf samples collected from inoculated or systemic leaves at different time points. hpi, hours post‐inoculation; dpi, days post‐inoculation. Results are representative of three independent experiments, with error bars indicating SD (n = 3) [file MPP-21-303-s003.docx]

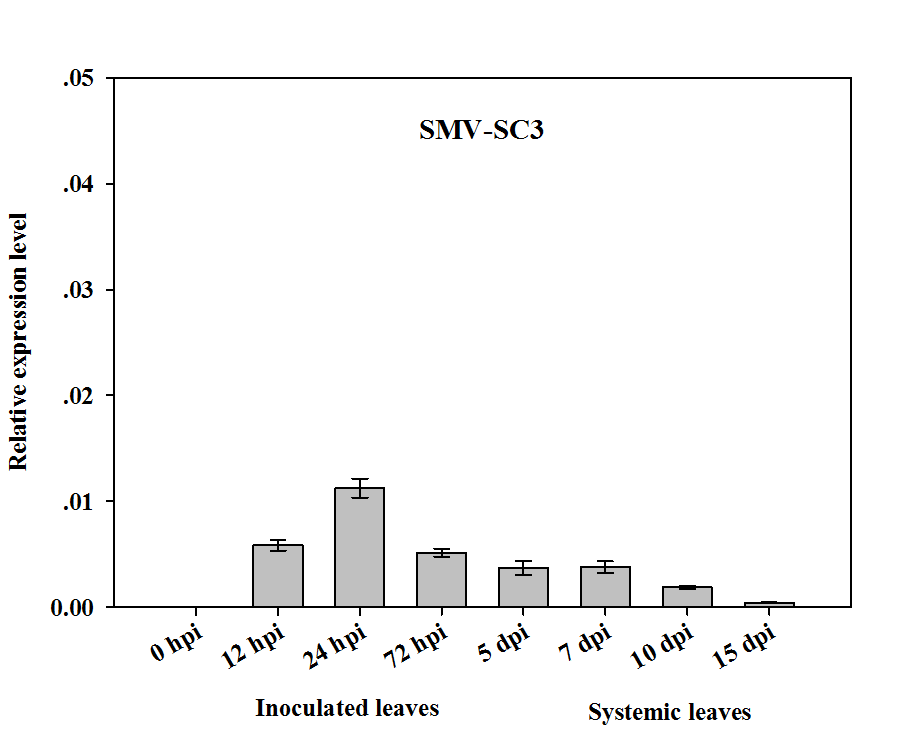


**Fig. S2** qRT-PCR detection of virus accumulation in Kefeng 1 after challenge with SMV strain SC3. Y-axis indicates SMV transcript levels. X-axis indicates leaf samples collected from inoculated or systemic leaves at different time points. hpi, hours post inoculation; dpi, days post inoculation. Results are representative of three independent experiments, with error bars indicating SD (n = 3).
