# Supplementary material for: Soybean RNA interference lines silenced for eIF4E show broad potyvirus resistance
Source: Mol Plant Pathol. 2019 Dec 20;21(3):303–17. doi: 10.1111/mpp.12897 (PMC7036369; doi:10.1111/mpp.12897)
Supplement: Supplementary file 4 — Fig S3 RT‐qPCR detection of the relative expression levels of soybean eIF4E1 and eIF4E2 in T5 plants derived from T0 line 1. The y axis indicates transcript levels of eIF4E1 and eIF4E2. The x axis indicates T5 and nontransformed (NT) plants. Results are representative of three independent experiments, with error bars indicating SD (n = 3) [file MPP-21-303-s004.docx]

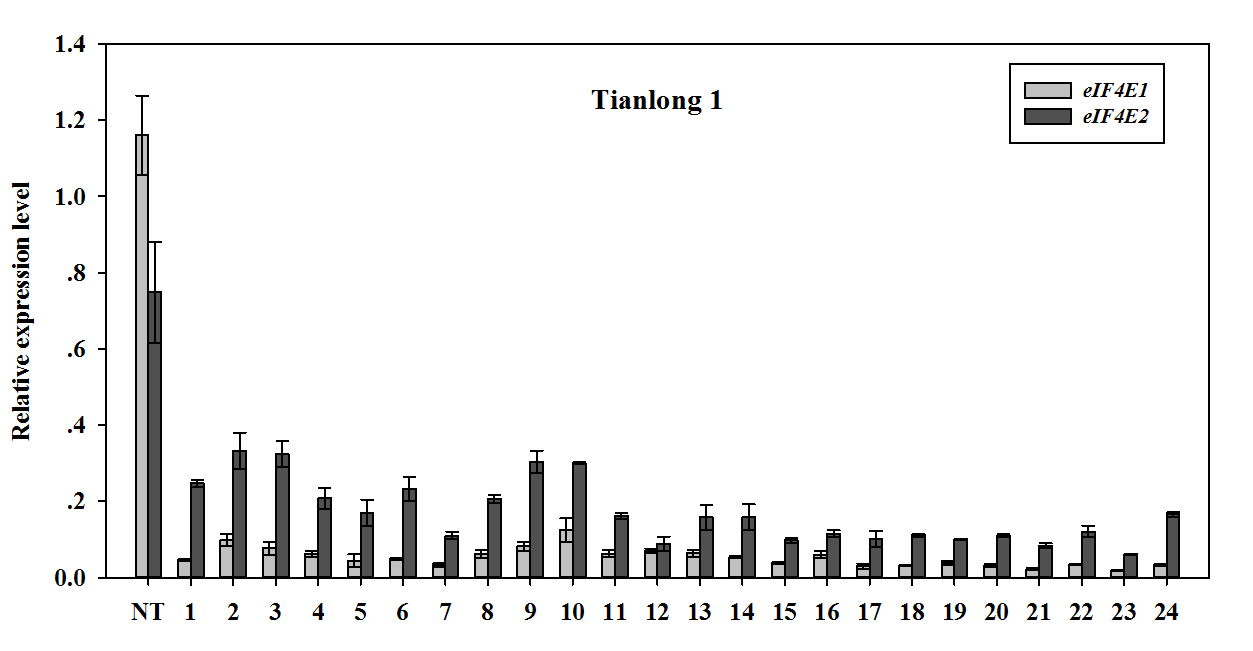


**Fig. S3** qRT-PCR detection of the relative expression levels of soybean *eIF4E1* and *eIF4E2* in T_5_ plants derived from T_0_ line 1. Y-axis indicates transcript levels of *eIF4E1* and *eIF4E2*. X-axis indicates T_5_ and nontransformed (NT) plants. Results are representative of three independent experiments, with error bars indicating SD (n = 3).
