# Supplementary material for: Soybean RNA interference lines silenced for eIF4E show broad potyvirus resistance
Source: Mol Plant Pathol. 2019 Dec 20;21(3):303–17. doi: 10.1111/mpp.12897 (PMC7036369; doi:10.1111/mpp.12897)
Supplement: Supplementary file 5 — Fig S4 Western blot analysis confirming the expression of fusion proteins in Nicotiana benthamiana for subcellular localization and bimolecular fluorescence complementation (BiFC) assay. (a) Fusion proteins for subcellular localization detected using green fluorescent protein (GFP) antibody. (b) Fusion proteins for BiFC detected using FLAG‐Tag antibody. (c) Fusion proteins for BiFC detected using HA‐Tag antibody. Positions of protein mobility markers in kilodaltons (kDa) are indicated on the left [file MPP-21-303-s005.docx]

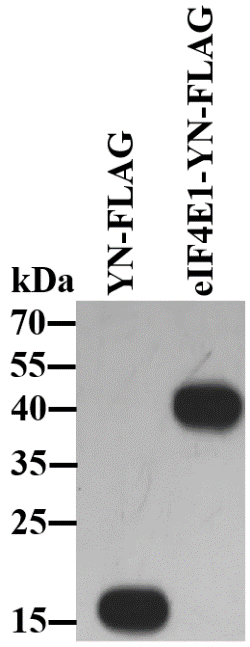

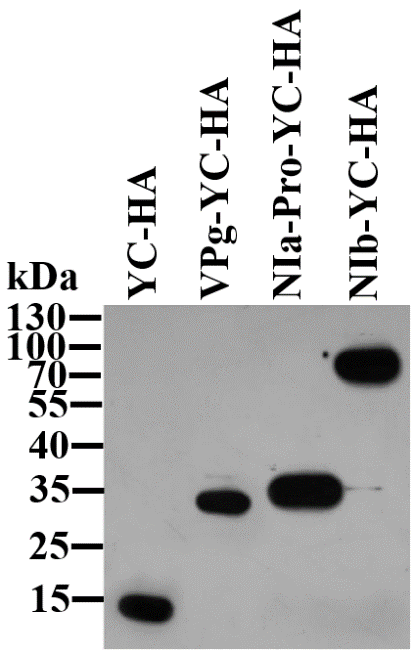


(b)

(c)

(a)


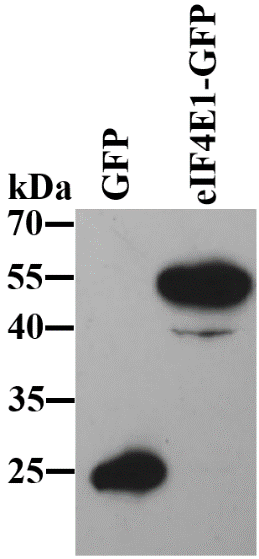


**Fig. S4** Western blot analysis confirming the expression of fusion proteins in *N. benthamiana* for subcellular localization and bimolecular fluorescence complementation (BiFC) assay. (a) Fusion proteins for subcellular localization detected using GFP antibody. (b) Fusion proteins for BiFC detected using FLAG-Tag antibody. (c) Fusion proteins for BiFC detected using HA-Tag antibody. Positions of protein mobility markers in kilodaltons (kDa) are indicated on the left.
