# Supplementary material for: Soybean RNA interference lines silenced for eIF4E show broad potyvirus resistance
Source: Mol Plant Pathol. 2019 Dec 20;21(3):303–17. doi: 10.1111/mpp.12897 (PMC7036369; doi:10.1111/mpp.12897)
Supplement: Supplementary file 6 — Table S1 Sequences of primer pairs used in this study [file MPP-21-303-s006.docx]

| Primer no. | Name | Forward primers (5′→3′) | Reverse primers (5′→3′) | Length (bp) | Purpose |
| --- | --- | --- | --- | --- | --- |
| 1 | *eIF4E1* | ATGGTTGTAGAAGATACCCAAAAGT | TACAACGTATTTATTTTTAGCACCTCT | 711 | Cloning of *eIF4E1* used for subcellular localization, Y2H and BiFC |
| 2 | *eIF4E1i* | CCCATCTACACTTTCGCCAC | CTGCTTTCCAATGCTCACCT | 348 | Cloning of *eIF4E1i* fragment used for RNAi vector construction |
| 3 | q*eIF4E1*-TL/KF | GTCGACGGCGAAGATAGCG | GCAGATAGGGTCCTCCCACT | 288 | qRT‑PCR for detecting the spatiotemporal expression profiles of *eIF4E1* |
| 4 | q*eIF4E1*-T_0_/T_5_ | GTCGACGGCGAAGATAGCG | CCCATGCGGCTTGTTTGGAC | 136 | qRT‑PCR for detecting the *eIF4E1* accumulation in T_0_/T_5_ plants |
| 5 | q*eIF4E2*-T_5_ | CGCCAACGACAACAACGA | GACGGAGGCTTTGATGTGG | 93 | qRT‑PCR for detecting the *eIF4E2* accumulation in T_5_ plants |
| 6 | q*Tubulin* | GGAGTTCACAGAGGCAGAG | CACTTACGCATCACATAGCA | 189 | Used as the internal reference control in qRT-PCR experiments |
| 7 | *bar* probe | GAGAATTAAGGGAGTCACGTTATG | CGTTGCGTGCCTTCCAG | 538 | Used for Southern blot hybridization analysis |
| 8 | SMV-*P1* | ATGGCAACAATCATGATTGGAA | GTAATGCTGAACATCCTCTATGTTTG | 927 | Cloning of SMV *P1* gene used for Y2H |
| 9 | SMV-*HC-Pro* | TCTCAAACTCCTGAAGCCCAGT | ACCAACTCTATAAAATTTCATCTCACTCT | 1371 | Cloning of SMV-*HC-Pro* gene used for Y2H |
| 10 | SMV-*P3* | GGGGAAGTGCAACAAAGGAT | CTGTGCGGAGACATCTTCTGAT | 1041 | Cloning of SMV *P3* gene used for Y2H |
| 11 | SMV-*P3N-PIPO* | GGGGAAGTGCAACAAAGGATGA | CATTTTTGGAAAAGTGTATTTCTTAC | 681 | Cloning of SMV *P3N-PIPO* gene used for Y2H |
| 12 | SMV-*6K1* | GCCAAAACAGCAACTCAATTG | CTGCACTTTAACATCCTCACCC | 156 | Cloning of SMV *6K1* gene used for Y2H |
| 13 | SMV-*CI* | AGTCTTGATGAGATTCAAAACATAGATG | CTGTAGTTGAACTGCATTTAAAATGC | 1902 | Cloning of SMV *CI* gene used for Y2H |
| 14 | SMV-*6K2* | AGCAAACATGAGGTGAGCAAAT | TTGAGTTGATACTGGCTCACGTATA | 159 | Cloning of SMV *6K2* gene used for Y2H |
| 15 | SMV-VPg | GGAAAGAAGAGGCAGATACAAAAA | TTCCATTTCAACCCTTTCTTTG | 570 | Cloning of SMV *VPg* gene used for Y2H and BiFC |
| 16 | SMV-*NIa-Pro* | AGCAAATCTGTCTACAAAGGACTTAG | TTGTACTGTCACTGTACTTCCAAAAAT | 729 | Cloning of SMV *NIa-Pro* gene used for Y2H and BiFC |
| 17 | SMV-*NIb* | GGGAAGAAGGAAAGATGGGTT | TTGTAAGGAAACTGATTCACAACAG | 1551 | Cloning of SMV *NIb* gene used for Y2H and BiFC |
| 18 | SMV-*CP* | TCAGGCAAGGAGAAAGAAGGA | CTGCTGTGGGCCCATGC | 795 | Cloning of SMV *CP* gene used for Y2H |
| 19 | qSMV-*CP* | CAGATGGGCGTGGTTATGA | ACAATGGGTTTCAGCGGATA | 128 | qRT‑PCR for detecting SMV accumulation in transgenic plants |
| 20 | qBCMV-*CP* | CCAACACTCCGCCAAATCAT | GCTGCCTTCATCTGTGCTAC | 209 | qRT‑PCR for detecting BCMV accumulation in transgenic plants |
| 21 | qWMV-*CP* | AGACAGGGAATTAGCACGCT | TTTACTGCGGTGGACCCATA | 236 | qRT‑PCR for detecting WMV accumulation in transgenic plants |
| 22 | qBPMV-*CP* | TGCTGTTCTTTCAATGTGGAGA | AGTGGACCATGCTGTGAGAA | 232 | qRT‑PCR for detecting BPMV accumulation in transgenic plants |

**Table S1** Sequences of primer pairs used in this study.
