# Supplementary material for: Soybean RNA interference lines silenced for eIF4E show broad potyvirus resistance
Source: Mol Plant Pathol. 2019 Dec 20;21(3):303–17. doi: 10.1111/mpp.12897 (PMC7036369; doi:10.1111/mpp.12897)
Supplement: Supplementary file 7 — Table S2 Efficiency of cotyledonary node‐Agrobacterium‐mediated soybean transformation. All positive plants were confirmed using leaf‐painting, PCR and LibertyLink strip. Transformation efficiency = (no. of positive T0 plants / no. of infected explants) × 100. Data was expressed as mean ± SD [file MPP-21-303-s007.docx]

**Table S2** Efficiency of cotyledonary node-*Agrobacterium*-mediated soybean transformation.

| Experiment no. | No. of infected explants | No. of regenerated plants | No. of positive T_0_ plants^a^ | Transformation efficiency (%)^b^ |
| --- | --- | --- | --- | --- |
| 1 | 287 | 4 | 4 | 1.39 |
| 2 | 212 | 10 | 7 | 3.30 |
| 3 | 209 | 12 | 8 | 3.83 |
| 4 | 259 | 6 | 3 | 1.16 |
| 5 | 230 | 6 | 5 | 2.17 |
| 6 | 191 | 8 | 4 | 2.09 |
| Total | 1388 | 46 | 31 | 2.23 ± 1.05^c^ |

^a^ All positive plants were confirmed using leaf-painting, PCR and LibertyLink^®^ strip.

^b^ Transformation efficiency = (No. of positive T_0_ plants / No. of infected explants) × 100.

^c^ Data was expressed as mean ± SD.
