# Supplementary material for: Soybean RNA interference lines silenced for eIF4E show broad potyvirus resistance
Source: Mol Plant Pathol. 2019 Dec 20;21(3):303–17. doi: 10.1111/mpp.12897 (PMC7036369; doi:10.1111/mpp.12897)
Supplement: Supplementary file 8 — Table S3 Investigation of seed coat mottling in T1–T4 lines after challenge with soybean mosaic virus (SMV) strain SC3. NT, nontransformed plant. Mottling rate = (total no. of mottled seeds / total no. of seeds) × 100 [file MPP-21-303-s008.docx]

**Table S3** Investigation of seed coat mottling in T_1_–T_4_ lines after challenge with SMV strain SC3.

| Generation | No. of plants evaluated | Total no. of seeds | Total no. of mottled seeds | Mottling rate (%)^a^ |
| --- | --- | --- | --- | --- |
| NT | 20 | 254 | 215 | 84.65 |
| T_1_ | 51 | 900 | 278 | 30.89 |
| T_2_ | 42 | 1324 | 42 | 3.17 |
| T_3_ | 42 | 1790 | 13 | 0.73 |
| T_4_ | 66 | 2910 | 40 | 1.37 |

NT, nontransformed plant.

^a^ Mottling rate = (Total no. of mottled seeds / Total no. of seeds) × 100.
