# Supplementary material for: Soybean RNA interference lines silenced for eIF4E show broad potyvirus resistance
Source: Mol Plant Pathol. 2019 Dec 20;21(3):303–17. doi: 10.1111/mpp.12897 (PMC7036369; doi:10.1111/mpp.12897)
Supplement: Supplementary file 9 — Table S4 DAS‐ELISA analysis of T2 plants inoculated with soybean mosaic virus (SMV) strain SC3. +, positive for SMV; ‐, negative for SMV; NT, nontransformed plant. OD405 value of each sample was calculated by averaging the three readings of the plate. OD405 value of negative control (mock inoculation) was calculated by averaging the three readings of the plate, which was 0.183 [file MPP-21-303-s009.docx]

**Table S4** DAS-ELISA analysis of T_2_ plants inoculated with SMV strain SC3.

| T_2_ plant no. | P^a^ (OD_405_) | P/N^b^ | T_2_ plant no. | P^a^ (OD_405_) | P/N^b^ |
| --- | --- | --- | --- | --- | --- |
| 1-1-1 | 0.192 | 1.05 (-) | 1-16-1 | 0.178 | 0.97 (-) |
| 1-1-2 | 0.142 | 0.77 (-) | 1-16-2 | 0.184 | 1.00 (-) |
| 1-1-3 | 0.164 | 0.90 (-) | 1-16-3 | 0.179 | 0.98 (-) |
| 1-1-4 | 0.188 | 1.03 (-) | 1-16-4 | 0.163 | 0.89 (-) |
| 1-1-5 | 0.166 | 0.90 (-) | 1-16-5 | 0.204 | 1.11 (-) |
| 1-1-6 | 0.196 | 1.07 (-) | 1-16-6 | 0.174 | 0.95 (-) |
| 1-1-7 | 0.169 | 0.92 (-) | 1-16-7 | 0.170 | 0.93 (-) |
| 1-1-8 | 0.183 | 1.00 (-) | 1-16-8 | 0.187 | 1.02 (-) |
| 1-1-9 | 0.176 | 0.96 (-) | 1-16-9 | 0.197 | 1.08 (-) |
| 1-1-10 | 0.154 | 0.84 (-) | 1-16-10 | 0.178 | 0.97 (-) |
| 1-1-11 | 0.207 | 1.13 (-) | 1-16-11 | 0.178 | 0.97 (-) |
| 1-1-12 | 0.185 | 1.01 (-) | 1-16-12 | 0.167 | 0.91 (-) |
| 1-1-13 | 1.590 | 8.68 (+) | 1-16-13 | 0.177 | 0.97 (-) |
| 1-1-14 | 0.192 | 1.05 (-) | 1-16-14 | 0.180 | 0.98 (-) |
| 1-1-15 | 0.187 | 1.02 (-) | 1-16-15 | 0.165 | 0.90 (-) |
| 1-1-16 | 1.779 | 9.71 (+) | 1-16-16 | 0.176 | 0.96 (-) |
| 1-1-17 | 0.210 | 1.15 (-) | NT | 2.584 | 14.11 (+) |
| 1-1-18 | 0.208 | 1.13 (-) |  |  |  |
| 1-1-19 | 0.186 | 1.02 (-) |  |  |  |
| 1-1-20 | 0.177 | 0.96 (-) |  |  |  |
| 1-1-21 | 0.189 | 1.03 (-) |  |  |  |
| 1-1-22 | 1.339 | 7.31 (+) |  |  |  |
| 1-1-23 | 0.186 | 1.02 (-) |  |  |  |
| 1-1-24 | 0.278 | 1.52 (-) |  |  |  |
| 1-1-25 | 0.190 | 1.04 (-) |  |  |  |
| 1-1-26 | 0.190 | 1.03 (-) |  |  |  |

+, positive for SMV; -, negative for SMV; NT, nontransformed plant.

^a^ OD_405_ value of each sample was calculated by averaging the three readings of the plate.

^b^ OD_405_ value of negative control (mock-inoculation) was calculated by averaging the three readings of the plate, which was 0.183.
