# Supplementary material for: Soybean RNA interference lines silenced for eIF4E show broad potyvirus resistance
Source: Mol Plant Pathol. 2019 Dec 20;21(3):303–17. doi: 10.1111/mpp.12897 (PMC7036369; doi:10.1111/mpp.12897)
Supplement: Supplementary file 10 — Table S5 DAS‐ELISA analysis of T2 lines inoculated with different viruses. SMV, soybean mosaic virus; BCMV, bean common mosaic virus; WMV, watermelon mosaic virus; BPMV, bean pod mottle virus; NT, nontransformed plant; wpi, weeks post‐inoculation; +, positive for virus; −, negative for virus. OD405 value of each T2 line was calculated by averaging the values of five T3 plants randomly selected from the line. OD405 value of each positive control was calculated by averaging the values of three virus‐inoculated NT plants, and OD405 value of each negative control was calculated by averaging the values of three mock‐inoculated NT plants [file MPP-21-303-s010.docx]

**Table S5** DAS-ELISA analysis of T_2_ lines inoculated with different viruses.

| Virus | T_2_ line no.^a^ | 3 wpi | 5 wpi | Virus | T_2_ line no.^a^ | 3 wpi | 5 wpi |
| --- | --- | --- | --- | --- | --- | --- | --- |
| SMV-SC3 | NT^b^ | >10 (+) | >10 (+) | SMV-SC18 | NT | >10 (+) | >10 (+) |
|  | 1-1-1 | 1.13 (-) | 1.03 (-) |  | 1-1-16 | 1.21 (-) | 1.57 (-) |
|  | 1-1-2 | 1.49 (-) | 1.61 (-) |  | 1-1-24 | 1.55 (-) | 1.69 (-) |
| SMV-SC7 | NT | >10 (+) | >10 (+) | SMV-R | NT | >10 (+) | >10 (+) |
|  | 1-1-4 | 1.09 (-) | 1.13 (-) |  | 1-16-4 | 1.13 (-) | 1.63 (-) |
|  | 1-1-9 | 1.05 (-) | 0.83 (-) | BCMV | NT | >10 (+) | >10 (+) |
|  | 1-16-5 | 0.92 (-) | 1.44 (-) |  | 1-16-10 | 1.38 (-) | 1.66 (-) |
| SMV-SC15 | NT | >10 (+) | >10 (+) | WMV | NT | >10 (+) | >10 (+) |
|  | 1-1-14 | 1.65 (-) | 1.46 (-) |  | 1-16-16 | 1.06(-) | 1.46 (-) |
|  | 1-1-19 | 0.90 (-) | 0.72 (-) | BPMV | NT | >10 (+) | >10 (+) |
|  |  |  |  |  | 1-16-6 | >10 (+) | >10 (+) |

SMV, soybean mosaic virus; BCMV, bean common mosaic virus; WMV, watermelon mosaic virus; BPMV, bean pod mottle virus; NT, nontransformed plant; wpi, weeks post inoculation; +, positive for virus; -, negative for virus.

^a^ OD_405_ value of each T_2_ line was calculated by averaging the values of five T_3_ plants randomly selected from the line.

^b^ OD_405_ value of each positive control was calculated by averaging the values of three virus-inoculated NT plants, and OD_405_ value of each negative control was calculated by averaging the values of three mock-inoculated NT plants.
