# Supplementary material for: Soybean RNA interference lines silenced for eIF4E show broad potyvirus resistance
Source: Mol Plant Pathol. 2019 Dec 20;21(3):303–17. doi: 10.1111/mpp.12897 (PMC7036369; doi:10.1111/mpp.12897)
Supplement: Supplementary file 11 — Table S6 DAS‐ELISA analysis of T3 lines inoculated with different viruses. SMV, soybean mosaic virus; BCMV, bean common mosaic virus; WMV, watermelon mosaic virus; BPMV, bean pod mottle virus; NT, nontransformed plant; wpi, weeks post‐inoculation; +, positive for virus; −, negative for virus. OD405 value of each T3 line was calculated by averaging the values of five T4 plants randomly selected from the line. OD405 value of each positive control was calculated by averaging the values of three virus‐inoculated NT plants, and OD405 value of each negative control was calculated by averaging the values of three mock‐inoculated NT plants [file MPP-21-303-s011.docx]

**Table S6** DAS-ELISA analysis of T_3_ lines inoculated with different viruses.

| Virus | T_3_ line no.^a^ | 3 wpi | 5 wpi | Virus | T_3_ line no.^a^ | 3 wpi | 5 wpi |
| --- | --- | --- | --- | --- | --- | --- | --- |
| SMV-SC3 | NT^b^ | >10 (+) | >10 (+) | SMV-SC18 | NT | >10 (+) | >10 (+) |
|  | 1-1-1-7 | 1.05 (-) | 0.96 (-) |  | 1-1-4-14 | 1.00 (-) | 1.01 (-) |
|  | 1-1-1-17 | 1.07 (-) | 1.15 (-) |  | 1-1-14-8 | 1.02 (-) | 1.32 (-) |
|  | 1-1-2-4 | 1.12 (-) | 1.28 (-) |  | 1-1-14-13 | 0.99 (-) | 1.08 (-) |
|  | 1-16-4-15 | 1.13 (-) | 1.22 (-) |  | 1-16-4-18 | 1.07 (-) | 1.10 (-) |
|  | 1-16-5-14 | 1.06 (-) | 0.97 (-) | SMV-R | NT | >10 (+) | >10 (+) |
| SMV-SC7 | NT | >10 (+) | >10 (+) |  | 1-16-4-3 | 1.02 (-) | 1.00 (-) |
|  | 1-1-16-1 | 1.29 (-) | 1.08 (-) |  | 1-16-4-12 | 1.08 (-) | 0.93 (-) |
|  | 1-1-16-5 | 1.05 (-) | 1.89 (-) | BCMV | NT | >10 (+) | >10 (+) |
|  | 1-1-19-8 | 0.98 (-) | 0.89 (-) |  | 1-16-2-9 | 0.96 (-) | 0.93 (-) |
|  | 1-1-24-1 | 1.05 (-) | 1.98 (-) |  | 1-16-2-12 | 1.05 (-) | 0.93 (-) |
|  | 1-16-6-3 | 1.04 (-) | 1.10 (-) | WMV | NT | >10 (+) | >10 (+) |
|  | 1-16-10-5 | 1.00 (-) | 1.06 (-) |  | 1-16-5-2 | 0.99 (-) | 1.07 (-) |
| SMV-SC15 | NT | >10 (+) | >10 (+) |  | 1-16-5-5 | 0.99 (-) | 1.20 (-) |
|  | 1-1-9-3 | 1.78 (-) | 1.12 (-) | BPMV | NT | >10 (+) | >10 (+) |
|  | 1-1-9-9 | 1.37 (-) | 1.69 (-) |  | 1-16-16-17 | >10 (+) | >10 (+) |
|  | 1-16-2-11 | 1.16 (-) | 1.49 (-) |  |  |  |  |
|  | 1-16-16-6 | 1.25 (-) | 1.26 (-) |  |  |  |  |

SMV, soybean mosaic virus; BCMV, bean common mosaic virus; WMV, watermelon mosaic virus; BPMV, bean pod mottle virus; NT, nontransformed plant; wpi, weeks post inoculation; +, positive for virus; -, negative for virus.

^a^ OD_405_ value of each T_3_ line was calculated by averaging the values of five T_4_ plants randomly selected from the line.

^b^ OD_405_ value of each positive control was calculated by averaging the values of three virus-inoculated NT plants, and OD_405_ value of each negative control was calculated by averaging the values of three mock-inoculated NT plants.
