# Supplementary material for: Soybean RNA interference lines silenced for eIF4E show broad potyvirus resistance
Source: Mol Plant Pathol. 2019 Dec 20;21(3):303–17. doi: 10.1111/mpp.12897 (PMC7036369; doi:10.1111/mpp.12897)
Supplement: Supplementary file 12 — Table S7 The 208 soybean cultivars used for SMV resistance assessment. SMV, soybean mosaic virus. Seventeen soybean cultivars identified as SMV‐resistant are highlighted in bold [file MPP-21-303-s012.docx]

**Table S7** 208 soybean cultivars used for SMV resistance assessment^a^.

| Serial no. | Cultivar | Serial no. | Cultivar | Serial no. | Cultivar | Serial no. | Cultivar |
| --- | --- | --- | --- | --- | --- | --- | --- |
| 1 | A3 | 53 | Zhongzuo 06-887 | 105 | Zhongzuo MG50727 | 157 | Zhou 02005 |
| 2 | **Zhongzuo 02-760** | 54 | Gui 605 | 106 | Zhou S06-365 | 158 | Zhongdou 33 |
| 3 | Zhe 98012 | 55 | **Zhongzuo 081** | 107 | Hujiao 07568 | 159 | You 07-76 |
| 4 | Yuechun 2010 | 56 | Zhongzuo J5050 | 108 | Yudou 22 | 160 | **Feicuixiabao** |
| 5 | Ji 008 | 57 | 03-5011-41 | 109 | Zhongdou 8 | 161 | Youchun 09-4 |
| 6 | Tongchun 1 | 58 | SD1019 | 110 | Han 4324 | 162 | Yundadou 11 |
| 7 | Zhongzuo 0670-72 | 59 | **BQ15** | 111 | K06-82 | 163 | Kfeng 75-3 |
| 8 | K070-2 | 60 | Jidou 17 | 112 | Fendou 85 | 164 | Zhe H0501 |
| 9 | Gui 0521-2 | 61 | **Kfeng 74-2** | 113 | Tie 01092-4 | 165 | Hongda 7 |
| 10 | Ji 098112 | 62 | **K06-47** | 114 | Zhongpin 03-6025 | 166 | Fuyou 6 |
| 11 | **Zhongzuo 06-06** | 63 | K05-36 | 115 | Jiusan 08 | 167 | Zhe 98010 |
| 12 | Quandou 12 | 64 | Zhongzuo 071 | 116 | Wupiqingren | 168 | Yushengdou 4 |
| 13 | W1 | 65 | Shangdou 15 | 117 | Ji 08B1 | 169 | Kfeng 75 |
| 14 | Zhe 98002 | 66 | Zhongzuo 042 | 118 | Hujiao 06-698 | 170 | Nannong 30 |
| 15 | Pudou 11 | 67 | Nannong 88-31 | 119 | Nannong 307 | 171 | Zhe 88005-3 |
| 16 | Ji 098098 | 68 | Gongqiudou 4 | 120 | Jinyi 50 | 172 | **Liao 04M05-3** |
| 17 | Ji 087120 | 69 | BQ12 | 121 | Lindou 9 | 173 | Hua 2 |
| 18 | Zhongzuo 07-754 | 70 | SN-zaohuang 2 | 122 | Jinda 78 | 174 | Qingsu 5 |
| 19 | Maodou 3 | 71 | K05-36 | 123 | Zhongzuo J5044 | 175 | SFGLV1 |
| 20 | Zhongpin 03-5027 | 72 | Fendou 79 | 124 | Gui 603 | 176 | Qiandou 1 |
| 21 | Zhongzuo 06-875 | 73 | Suike 8 | 125 | BQ16 | 177 | Zhe H0427 |
| 22 | Kuixian 1 | 74 | BQ18 | 126 | Zhongzuo 06-06 | 178 | Lvbaoshi |
| 23 | Zhongzuo J7018 | 75 | AQ04 | 127 | Dian 86-5 | 179 | Wuqing 2 |
| 24 | Zhongzuo 075253 | 76 | Xiangchundou 24 | 128 | Zhonghuang 26 | 180 | Hongqiuguifei |
| 25 | Gui H49 | 77 | Ke 9302 | 129 | Zhe H0501 | 181 | Kaiyu 9 |
| 26 | Zhongzuo J8024 | 78 | Nannong 307 | 130 | D8 | 182 | AGS292 |
| 27 | Huachun 6 | 79 | 20113-3 | 131 | Fu 08-238 | 183 | Edou 010 |
| 28 | A1 | 80 | Lu 96150 | 132 | Jiu 658 | 184 | Dian 86-5 |
| 29 | **Zhongzuo J8035** | 81 | B5 | 133 | Zhongzuo A054 | 185 | Zhe H0431 |
| 30 | Kuixian 2 | 82 | Zhongzuo J8149 | 134 | Zhongzuo J8033 | 186 | Zhe A8901 |
| 31 | **A2** | 83 | Quandou 11 | 135 | Qihuang 33 | 187 | Qiandou 08003 |
| 32 | **Liao 03M02** | 84 | Gongjiao 999221 | 136 | Shidou 101 | 188 | Zhexiandou 5 |
| 33 | Taiwan 75 | 85 | Zheng 3074 | 137 | Zhongzuo 07-22 | 189 | Hongqiu 88 |
| 34 | ACK | 86 | CQ24 | 138 | Handou 5 | 190 | An 08019 |
| 35 | **Liao 00128** | 87 | C2 | 139 | YN-59 | 191 | Gongdou 723-2 |
| 36 | Ji 098113 | 88 | Jinli 8 | 140 | Zhongpin 03-6025 | 192 | Zhenlong 2 |
| 37 | **KF 146-7** | 89 | Hujiao 03-263 | 141 | BQ11 | 193 | Zhe H0428-25 |
| 38 | Zhongzuo J8023 | 90 | Zhongzuo J9331 | 142 | Hedou 12 | 194 | Edou 012 |
| 39 | Lvbaozhu CK1 | 91 | Guichun 11 | 143 | **Zhongzuo 05-675** | 195 | Jipintianjian |
| 40 | A4 | 92 | Gongjiao H041-22 | 144 | Shi H570 | 196 | Shuxian 205 |
| 41 | BN 105 | 93 | CQ21 | 145 | Zhe H0431 | 197 | Tianlong 1 |
| 42 | Zhe 087201 | 94 | L-6 | 146 | Zhonghuang 46 | 198 | Jing 0-51 |
| 43 | Zhe H0526 | 95 | Qu 9887 | 147 | Gongdou 378 | 199 | Nannong D1 |
| 44 | Taiwan 75-2 | 96 | Xu 99016 | 148 | Zhe 8805-7 | 200 | Kfeng 75-2 |
| 45 | **Zhonghuang 39-2** | 97 | SD1008 | 149 | AQ03 | 201 | Diancang 2 |
| 46 | Zhonghuang 39 | 98 | Zhongzuo 049 | 150 | Fu 0890 | 202 | **Zhenlong 5** |
| 47 | Zhengxian 1 | 99 | K07SP | 151 | Gongjiao H04-71 | 203 | **Jingfengzhizun** |
| 48 | Quandou 7 | 100 | Diandou 1 | 152 | Jidou 12 | 204 | Xiangchundou 26 |
| 49 | Zhenong 0912 | 101 | Zhongzuo J9350 | 153 | Zhengdou EH009 | 205 | Nan F7256-3 |
| 50 | Zhongzuo 042 | 102 | Pudou 0656 | 154 | Zhe H0528 | 206 | K74-3 |
| 51 | Yuechun 2010-2 | 103 | SD1020 | 155 | HF | 207 | Jiaoda 08-26 |
| 52 | Tongchun 2 | 104 | HF-6 | 156 | Wuqing 1 | 208 | Nandou 12 |

SMV, soybean mosaic virus_._

^a^ Seventeen soybean cultivars identified as SMV-resistant are highlighted in bold.
