# Supplementary material for: Soybean RNA interference lines silenced for eIF4E show broad potyvirus resistance
Source: Mol Plant Pathol. 2019 Dec 20;21(3):303–17. doi: 10.1111/mpp.12897 (PMC7036369; doi:10.1111/mpp.12897)
Supplement: Supplementary file 13 — Table S8 Sequencing analysis of eIF4E1 from the 17 SMV‐resistant soybean cultivars and protein–protein interactions between mutated eIF4E1s and SMV VPg via Y2H. D, aspartic acid; H, histidine; K, lysine; N, asparagine; R, arginine; SMV, soybean mosaic virus; VPg, viral genome‐linked protein; Y2H, yeast two‐hybrid; +, interaction with SMV VPg; −, no interaction with SMV VPg. All mutations were compared with the soybean cultivar Nannong 1138‐2 (highly susceptible host) [file MPP-21-303-s013.docx]

**Table S8** Sequencing analysis of *eIF4E1* from the 17 SMV-resistant soybean cultivars and protein-protein interactions between mutated eIF4E1s and SMV VPg via Y2H^a^.

| Cultivar | No. of nucleotide mutation | No. of amino acid mutation | Amino acid mutation | Interaction with SMV VPg |
| --- | --- | --- | --- | --- |
| Zhongzuo 02-760 | 5 | 1 | D_508_→N_508_ | - |
| Zhongzuo 06-06 | 5 | 1 | D_508_→N_508_ | - |
| Zhongzuo J8035 | 2 | 1 | R_539_→K_539_ | - |
| A2 | 3 | 0 |  |  |
| Liao 03M02 | 3 | 0 |  |  |
| Liao 00128 | 3 | 0 |  |  |
| KF 146-7 | 0 | 0 |  |  |
| Zhonghuang 39-2 | 0 | 0 |  |  |
| Zhongzuo 081 | 0 | 0 |  |  |
| BQ15 | 2 | 1 | D_91_→H_91_ | + |
| Kfeng 74-2 | 0 | 0 |  |  |
| K06-47 | 0 | 0 |  |  |
| Zhongzuo 05-675 | 0 | 0 |  |  |
| Feicuixiabao | 0 | 0 |  |  |
| Liao 04M05-3 | 4 | 1 | R_539_→K_539_ | - |
| Zhenlong 5 | 0 | 0 |  |  |
| Jingfengzhizun | 0 | 0 |  |  |

D, aspartic acid; H, histidine; K, lysine; N, asparagine; R, arginine; SMV, soybean mosaic virus; VPg, viral genome-linked protein; Y2H, yeast two-hybrid; +, interaction with SMV VPg; -, no interaction with SMV VPg.

^a^ All the mutations were compared with the soybean cultivar Nannong 1138-2 (highly susceptible host).
